# Effect of High-Titer Convalescent Plasma on Progression to Severe Respiratory Failure or Death in Hospitalized Patients With COVID-19 Pneumonia: A Randomized Clinical Trial

**DOI:** 10.1001/jamanetworkopen.2021.36246

**Published:** 2021-11-29

**Authors:** Francesco Menichetti, Patrizia Popoli, Maria Puopolo, Stefania Spila Alegiani, Giusy Tiseo, Alessandro Bartoloni, Giuseppe Vittorio De Socio, Sauro Luchi, Pierluigi Blanc, Massimo Puoti, Elena Toschi, Marco Massari, Lucia Palmisano, Giuseppe Marano, Margherita Chiamenti, Laura Martinelli, Silvia Franchi, Carlo Pallotto, Lorenzo Roberto Suardi, Barbara Luciani Pasqua, Marco Merli, Plinio Fabiani, Luca Bertolucci, Beatrice Borchi, Sara Modica, Sara Moneta, Giulia Marchetti, Antonella d’Arminio Monforte, Laura Stoppini, Nadia Ferracchiato, Stefania Piconi, Claudio Fabbri, Enrico Beccastrini, Riccardo Saccardi, Andrea Giacometti, Sara Esperti, Piera Pierotti, Laura Bernini, Claudia Bianco, Sara Benedetti, Alessandra Lanzi, Paolo Bonfanti, Marco Massari, Spartaco Sani, Annalisa Saracino, Antonella Castagna, Luigia Trabace, Maria Lanza, Daniele Focosi, Alessandro Mazzoni, Mauro Pistello, Marco Falcone

**Affiliations:** 1 Infectious Disease Unit, Department of Clinical and Experimental Medicine, Azienda Ospedaliera Universitaria Pisana, University of Pisa, Pisa, Italy; 2 National Center for Drug Research and Evaluation, Istituto Superiore di Sanità, Rome, Italy; 3 Department of Neuroscience, Istituto Superiore di Sanità, Rome, Italy; 4 Infectious and Tropical Diseases Unit, Florence Department of Medicine, Careggi University Hospital, Florence, Italy; 5 Clinic of Infectious Diseases, “Santa Maria della Misericordia” Hospital, University of Perugia, Perugia, Italy; 6 Infectious Disease Unit, Hospital of Lucca, Lucca, Italy; 7 Infectious Diseases, Ospedale S. Maria Annunziata, Firenze, Italy; 8 University of Milano-Bicocca School of Medicine, Milan, Italy; 9 Azienda socio sanitaria territorial (ASST) Grande Ospedale Metropolitano Niguarda, Milan, Italy; 10 Research Coordination and Support Service (CoRi), Istituto Superiore di Sanità, Rome, Italy; 11 Italian National Blood Centre, Rome, Italy; 12 Unit of Infectious Diseases, Carlo Poma Hospital, Mantova, Italy; 13 Internal Medicine, Unità Sanitaria Locale (USL)–Umbria 1, Ospedale Città di Castello, Città di Castello, Italy; 14 Infectious Diseases Unit, San Giuseppe Hospital, Azienda USL Toscana Centro, Empoli, Italy; 15 Centro Regionale Sangue, Servizio Immunotrasfusionale, Azienda Ospedaliera di Perugia, Perugia, Italy; 16 Internal Medicine, Ospedale Unico della Versilia, Lido di Camaiore, Italy; 17 Infectious Diseases Unit, Department of Health Sciences, ASST Santi Paolo e Carlo University Hospital, Milan, Italy; 18 Internal Medicine, Ospedale di Foligno, Foligno, Italy; 19 Infectious Diseases, Azienda Ospedaliera di Lecco, Lecco, Italy; 20 Infectious Diseases, Ospedale San Jacopo, Pistoia, Italy; 21 Cell Therapy and Transfusion Medicine, Careggi University Hospital, Florence, Italy; 22 Azienda Ospedaliera Universitaria, Ospedali Riuniti di Ancona, Ancona, Italy; 23 Division of Infectious Diseases, Arezzo Hospital, Arezzo, Italy; 24 Department of Infectious Diseases, ASST Monza, University of Milano-Bicocca, Milan, Italy; 25 Infectious Disease Unit, Azienda USL–Istituto di Ricovero e Cura a Carattere Scientifico (IRCCS) di Reggio Emilia, Reggio Emilia, Italy; 26 Infectious Diseases, Livorno Hospital, Livorno, Italy; 27 Division of Infectious Diseases, Bari University Hospital, Bari, Italy; 28 Infectious Diseases, IRCCS Ospedale San Raffaele, Università Vita-Salute San Raffaele, Milan, Italy; 29 Department of Experimental and Clinical Medicine, University of Foggia, Foggia, Italy; 30 North-Western Tuscany Blood Bank, Pisa University Hospital, Pisa, Italy; 31 Division of Transfusion Medicine and Transplant Biology, Pisa University Hospital, Pisa, Italy; 32 Division of Virology, University Hospital of Pisa, Retrovirus Center, Department of Translational Research, University of Pisa, Pisa, Italy

## Abstract

**Importance:**

Convalescent plasma (CP) has been generally unsuccessful in preventing worsening of respiratory failure or death in hospitalized patients with COVID-19 pneumonia.

**Objective:**

To evaluate the efficacy of CP plus standard therapy (ST) vs ST alone in preventing worsening respiratory failure or death in patients with COVID-19 pneumonia.

**Design, Setting, and Participants:**

This prospective, open-label, randomized clinical trial enrolled (1:1 ratio) hospitalized patients with COVID-19 pneumonia to receive CP plus ST or ST alone between July 15 and December 8, 2020, at 27 clinical sites in Italy. Hospitalized adults with COVID-19 pneumonia and a partial pressure of oxygen–to–fraction of inspired oxygen (Pao_2_/Fio_2_) ratio between 350 and 200 mm Hg were eligible.

**Interventions:**

Patients in the experimental group received intravenous high-titer CP (≥1:160, by microneutralization test) plus ST. The volume of infused CP was 200 mL given from 1 to a maximum of 3 infusions. Patients in the control group received ST, represented by remdesivir, glucocorticoids, and low–molecular weight heparin, according to the Agenzia Italiana del Farmaco recommendations.

**Main Outcomes and Measures:**

The primary outcome was a composite of worsening respiratory failure (Pao_2_/Fio_2_ ratio <150 mm Hg) or death within 30 days from randomization.

**Results:**

Of the 487 randomized patients (241 to CP plus ST; 246 to ST alone), 312 (64.1%) were men; the median (IQR) age was 64 (54.0-74.0) years. The modified intention-to-treat population included 473 patients. The primary end point occurred in 59 of 231 patients (25.5%) treated with CP and ST and in 67 of 239 patients (28.0%) who received ST (odds ratio, 0.88; 95% CI, 0.59-1.33; *P* = .54). Adverse events occurred more frequently in the CP group (12 of 241 [5.0%]) compared with the control group (4 of 246 [1.6%]; *P* = .04).

**Conclusions and Relevance:**

In patients with moderate to severe COVID-19 pneumonia, high-titer anti–SARS-CoV-2 CP did not reduce the progression to severe respiratory failure or death within 30 days.

**Trial Registration:**

ClinicalTrials.gov Identifier: NCT04716556

## Key Points

**Question:**

Is convalescent plasma useful in preventing worsening respiratory failure or death in patients with COVID-19 pneumonia?

**Findings:**

In this randomized clinical trial of 487 patients with COVID-19 pneumonia and a partial pressure of arterial oxygen–to–fraction of inspired oxygen (Pao_2_/Fio_2_) ratio between 350 and 200 mm Hg at enrollment, the rate of the primary clinical end point (need for mechanical ventilation, defined as Pao_2_/Fio_2_ ratio <150 mm Hg, or death) was not significantly different between the convalescent plasma group and the control group.

**Meaning:**

In this trial, convalescent plasma did not reduce the progression to severe respiratory failure or death within 30 days.

## Teaser

This randomized clinical trial evaluates the efficacy of convalescent plasma plus standard therapy vs standard care alone in preventing worsening respiratory failure or death in patients with COVID-19 pneumonia.

## Introduction

Convalescent plasma (CP) from individuals recovered from SARS-CoV-2 infection has been proposed as therapy for patients with COVID-19, but the clinical evidence of its benefit is limited. Randomized clinical trials (RCTs) already published^1,2,3,4,5,6,7,8,9^ or available as preprint versions^10,11,12^ have not shown a clear benefit of CP in reducing the risk of disease progression or death. However, a relationship between neutralizing antibody (NAb) titer and a more favorable clinical outcome have been suggested,^9,13^ and CP was associated with a decreased 28-day mortality rate when high titer plasma was used^14^ or when CP was administered early in the course of the disease.^15^ Data from available RCTs have several limitations, including the administration of plasma with low NAb titer, the use of suboptimal surrogate serological tests to determine NAb titer, and most importantly, the delayed administration of CP from the onset of COVID-19 symptoms. The aim of this study was to evaluate the efficacy of high-titer CP in patients with SARS-CoV-2 pneumonia.

## Methods

### Trial Design

We performed a multicenter, national, randomized, open-label, controlled trial between July 15 and December 8, 2020, at 27 clinical sites in Italy (the Transfusion of Convalescent Plasma for the Early Treatment of Patients With COVID-19 [TSUNAMI] trial). The trial was sponsored by the Italian National Institute of Health (Istituto Superiore di Sanità [ISS]) and by the Italian Medicines Agency (Agenzia Italiana del Farmaco [AIFA]). The study protocol has been reviewed and approved by the ethical committee of the National Institute for Infectious Diseases, L. Spallanzani (Supplement 1). The Gruppo Italiano Malattie Ematologiche dell’Adulto (GIMEMA) managed the randomization of patients and the database of the study through the creation of electronic case report forms on the REDCap secure web application hosted by GIMEMA itself. In addition, GIMEMA performed the monitoring activity.^16^ The trial was coordinated by an ad hoc group established at the ISS and supervised by an independent data and safety monitoring board. The trial was conducted in accordance with the principles stated in the Declaration of Helsinki^17^ and Good Clinical Practices guidelines. This study followed the Consolidated Standards of Reporting Trials (CONSORT) reporting guideline for randomized clinical trial.

### Inclusion and Exclusion Criteria

Patients aged at least 18 years with confirmed COVID-19 based on a positive reverse transcriptase–polymerase chain reaction (RT-PCR) result for SARS-CoV-2 admitted to the participating clinical sites were screened for eligibility. Inclusion criteria were radiologically confirmed pneumonia within no more than 10 days from onset of symptoms and partial pressure of oxygen–to–fraction of inspired oxygen (Pao_2_/Fio_2_) ratio between 200 and 350 mm Hg at baseline. We excluded pregnant and lactating women, patients with known hypersensitivity to blood products, recipients of immunoglobulin in the past 30 days, patients with conditions precluding infusion of blood products, participants in any other clinical trials, and patients requiring noninvasive or invasive mechanical ventilation as well as patients receiving treatment with interleukin (IL) 1, IL 6, or Janus kinase inhibitors at the time of randomization. All participants or their family members or legally authorized representatives were provided with information about the trial in a language with which they were familiar, and written informed consent was obtained before recruitment.

Severity of pneumonia was assessed by the Pao_2_/Fio_2_ ratio. Race and ethnicity were collected from the medical record and self-reported by each patient. Race was then reported in the electronic case report form in the following categories: Asian, Black or African American, and White.^18^ These variables were included to evaluate the need for adjustment between the study groups and among different sites.

### Randomization and Intervention

A stratified permuted block randomization procedure with a 1:1 ratio, with blocks of variable sizes and stratification for clinical sites, was generated by using Stata version 16.1 (StataCorp). Eligible patients underwent web-based treatment allocation through REDcap platform to receive either administration of COVID-19 CP in addition to standard therapy (ST) or ST alone. The volume of infused CP was 200 mL, given over a period of 2.0 hours daily from 1 to a maximum of 3 infusions. ST was represented by remdesivir (intravenous [IV], 200 mg on the first day and 100 mg once daily from day 2 to day 5), glucocorticoids (IV dexamethasone 6 mg daily or equivalent), and low–molecular weight heparin (subcutaneous enoxaparin, 40-60 mg daily or intermediate/high dose in selected cases), according to the AIFA recommendations.^19^

### Plasma Collection and Preparation

Eligible donors were men or nulliparous women (aged 18 to 65 years), weighing more than 50 kg, with a previous diagnosis of COVID-19 confirmed by a RT-PCR test. Donors had to be asymptomatic for at least 28 consecutive days before donation with 2 negative RT-PCR test results for SARS-CoV-2 from nasopharyngeal swabs collected 24 hours apart. Donors gave written informed consent before donation. CP units (volume approximately 600 mL) were collected when anti–SARS-CoV-2 microneutralization test (MNT) showed an antibody titer of at least 1:160. MNT was performed in VeroE6-cells.^20^ Plasma was inactivated with a pathogen reduction technology (either Intercept or Mirasol),^21^ aliquoted in three 200-mL subunits, and either frozen or freshly distributed. Frozen subunits were thawed on demand and transfused within 5 days. All routine screening tests, including ABO blood grouping; Rhesus phenotype; complete blood counts; screening for HIV, hepatitis B or C virus, syphilis, and total serum protein were conducted according to the recommendation of the Italian National Blood Center.^22^

### Trial End Points

Primary and secondary outcomes were assessed during a follow-up period lasting 30 days from randomization. The primary end point was a composite of worsening respiratory failure (defined as a Pao_2_/Fio_2_ ratio <150 mm Hg), indicating the potential need for mechanical ventilation, or death. Prespecified secondary clinical end points were 30-day mortality; mechanical ventilation or death; virological clearance (defined by 2 consecutive negative nasopharyngeal swabs performed at least 24 hours apart); and length of hospital stay (days). Data about primary and secondary outcomes were collected by each local subinvestigator. Clinical evaluation of patients was performed daily, while nasopharyngeal swabs, laboratory examinations, and blood gas analysis (for the calculation of the Pao_2_/Fio_2_) were performed on days 1, 3, 7, 14, and 30 after randomization, unless clinically indicated according to physician judgment. If the patient was discharged before 30 days since randomization, telephone visits were performed on day 30 (±1 day). The incidence of adverse events (AEs) was analyzed. AEs included fever, transfusion-related acute lung injury, transfusion-associated circulatory overload, allergic/anaphylactic transfusion reactions, and hemolytic transfusion reactions.

### Statistical Analysis

Statistical analyses were conducted in accordance with the predefined statistical analysis plan. The intention-to-treat (ITT) population included randomized patients, except for those who withdrew informed consent (eAppendix in Supplement 2). Efficacy analyses were based on the modified ITT (mITT), which included patients who received allocated treatment, and per-protocol (PP) populations, whereas safety analyses were conducted on the randomized population. Data were expressed as means and SDs if normally distributed and medians and ranges or IQRs if not normally distributed. The Mann-Whitney test was used for continuous data; frequencies, percentages, and the χ^2^ test was used for categorical variables; Kaplan-Meier time-to-event analyses were performed only for the secondary outcomes. Log-rank test for time-to-event data were calculated. The effect of CP and ST compared with ST was reported as odds ratios (ORs) and 95% CIs, estimated by univariate logistic regression models. Prespecified subgroup analyses on the primary end point were planned, and differences among subgroups were assessed through test of interaction terms in logistic regression models^23^ (eAppendix in Supplement 2). Tests were carried out at 2-sided *P* < .05 level of significance. The analyses were performed using Stata version 16.1 (StataCorp). By assuming that 30% of COVID-19 patients receiving ST would meet the composite primary outcome within 30 days from randomization,^24^ to show a clinically significant reduction of 40% in the CP plus ST group vs the ST group in the primary composite end point, with a power of 80% and significance level of 5%, a sample size of 237 participants in each group (total of 474 participants for the study) was estimated. Monitoring during the study was performed to avoid missing data in the primary analysis.

## Results

### Trial Population

**Figure 1.**
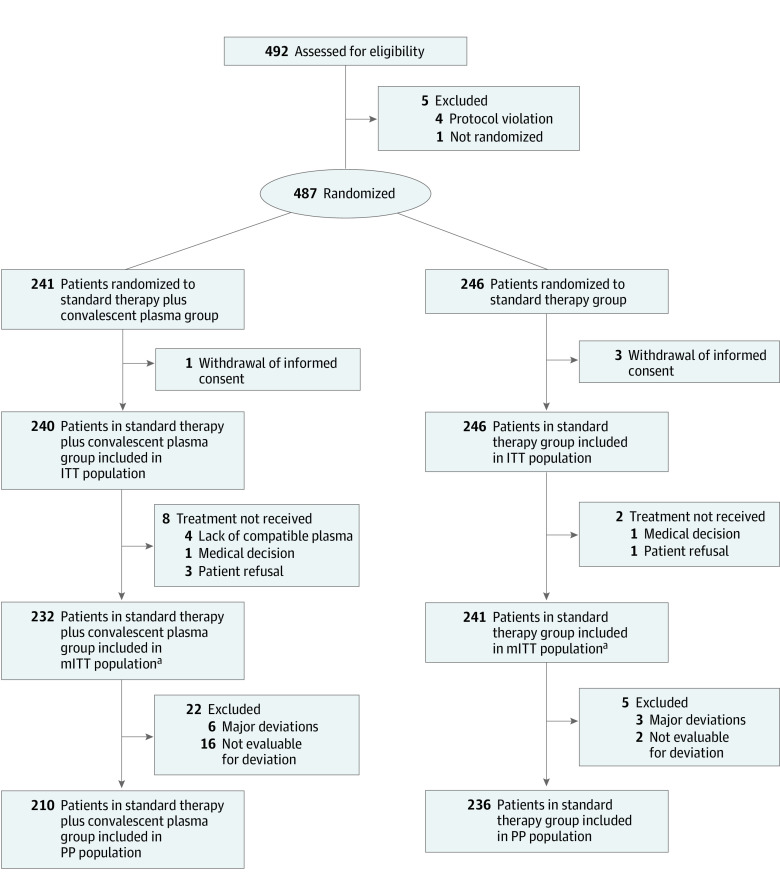
Study Flowchart Abbreviations: ITT, intention to treat population; mITT, modified intention to treat population; PP, per protocol population. ^a^Data not available for 3 patients (1 in convalescent plasma plus standard therapy group; 2 in standard therapy group).

In the study period, 487 patients underwent randomization. Of these, 241 were assigned to receive CP plus ST and 246 to ST alone. Median (IQR) age was 64 (54.0-74.0) years (65 [55-74] years in the CP plus ST group; 63.5 [54-74] years in the ST group). Overall, 312 patients (64.1%) were men (154 [63.9%] in the CP plus ST group and 158 [64.2%] in the ST group) (eTable 1 in Supplement 2). A total of 483 patients (240 for CP plus ST group and 243 for ST group) were included in the ITT population. The mITT population included 473 patients, 232 in the CP plus ST group and 241 in the ST group. The PP population included 446 patients, 210 in the CP plus ST and 236 in the ST group. The study flow is reported in Figure 1. Baseline characteristics of patients included in the mITT population did not differ among the trial group with respect to age, sex, ethnicity, body mass index, time from initial symptoms to randomization, comorbidities, baseline Pao_2_/Fio_2_ ratio, Sequential Organ Failure Assessment score, and previous treatments (Table 1). The median (IQR) time from onset of symptoms to CP infusion was 7.7 (5.0-9.0) days. A single CP infusion was administered to 35 of 232 patients (15.1%), 2 infusions to 175 (75.4%), and 3 infusions to 22 (9.5%). The median (IQR) NAb titer of the administered CP was 226.3 (160-320) (eTable 2 in Supplement 2); 149 patients (64.2%) received CP units with NAb titer less than 320, while 83 (35.8%) received CP units with NAb titers of at least 320. Anti-spike IgG antibodies at baseline were available for the 252 patients (53.3%) in the mITT population. Positive serology was detected in 28 of 112 patients (25.0%) and 31 of 140 patients (22.6%) in the CP plus ST and ST groups, respectively. Detailed data about oxygen supplementation and concomitant treatments from day 1 to 30 after randomization are reported in eTable 3 in Supplement 2.

**Table 1.** Patient Characteristics at the Baseline in the Modified Intention-to-Treat Population

Characteristic	Patients, No. (%)		
	Total (N = 473)^a^	Convalescent plasma plus standard therapy (n = 232)	Standard therapy (n = 241)
Age, median (IQR), y	64.0 (54.0-74.0)	65.0 (55.0-74.0)	63.0 (54.0-74.0)
Sex			
Female	169 (35.7)	82 (35.3)	87 (36.1)
Male	304 (64.3)	150 (64.7)	154 (63.9)
Race			
White	452 (95.6)	221 (95.3)	231 (92.8)
Black or African American	13 (2.7)	6 (2.6)	7 (2.9)
Asian	8 (1.7)	5 (2.2)	3 (1.2)
BMI, median (IQR)	26.0 (24.0-28.5)	26.0 (24.2-29.0)	26.0 (23.9-28.1)
Time from onset of symptoms to randomization, median (IQR), d	7.0 (5.0-9.0)	7.0 (5.0-9.0)	7.0 (4.0-9.0)
Coexisting conditions			
No comorbidities	98 (20.7)	48 (20.7)	50 (20.7)
Hypertension	179 (37.8)	82 (35.3)	97 (40.3)
Type 2 diabetes	91 (19.2)	46 (19.8)	45 (18.7)
COPD	27 (5.7)	13 (5.6)	14 (5.8)
Chronic kidney failure	22 (4.7)	6 (2.6)	16 (6.6)
Solid tumors	17 (3.6)	10 (4.3)	7 (2.9)
Congestive heart failure	11 (2.3)	5 (2.2)	6 (2.5)
Disease severity, median (IQR)			
Pao_2_/Fio_2_, mm Hg	272.5 (238.0-308.0)	277.0 (236.0-306.0)	266.0 (240.0-309.0)
SOFA score	2 (2-3)	2 (2-2)	2 (2-3)
Previous treatments			
Remdesivir	13 (2.7)	5 (2.2)	8 (3.3)
Glucocorticoids	97 (20.5)	45 (19.4)	52 (21.6)
LMWH	92 (19.5)	46 (19.8)	46 (19.1)

Abbreviations: BMI, body mass index (calculated as weight in kilograms divided by height in meters squared); COPD, chronic obstructive pulmonary disease; LMWH, low–molecular weight heparin; Pao_2_/Fio_2_, partial pressure of arterial oxygen to fraction of inspired oxygen; SOFA, Sequential Organ Failure Assessment.

^a^ Missing data for 106 patients (50 in convalescent plasma plus standard therapy group; 56 in standard therapy group). Continuous data (ie, age, BMI, time from onset of symptoms, Pao_2_/Fio_2_, and SOFA score) were not normally distributed (Shapiro-Wilk tests, *P* < .006).

### Primary End Point

**Figure 2.**
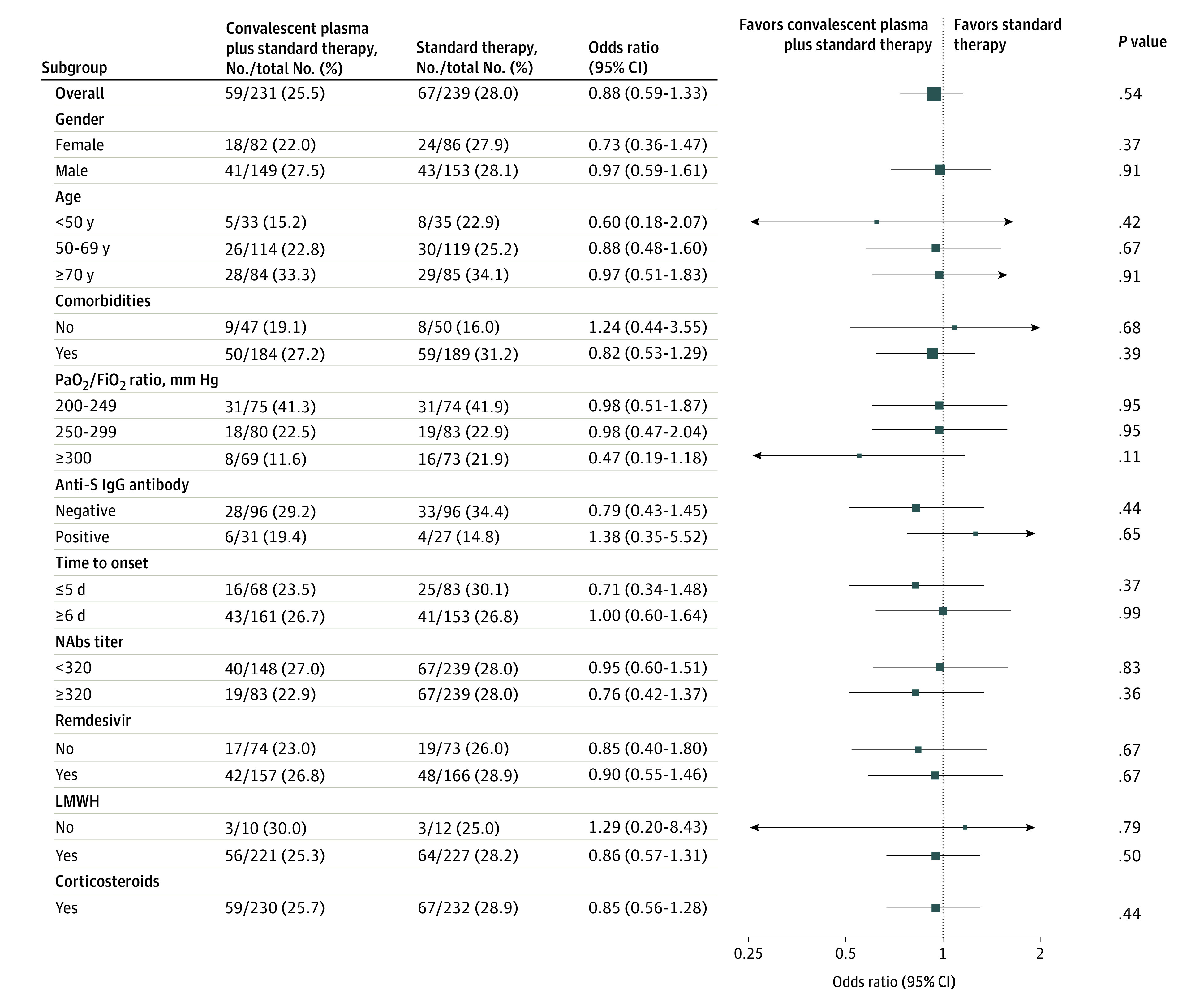
Primary End Point in the Modified Intention-to-Treat Population, Subgroup Analysis The primary end point was worsening respiratory failure or death within 30 days from randomization. Only 1 patient in the convalescent plasma (CP) plus standard therapy (ST) group and 7 in the ST group did not receive corticosteroids. LMWH indicates low–molecular weight heparin; NAb, neutralizing antibody.

**Figure 3.**
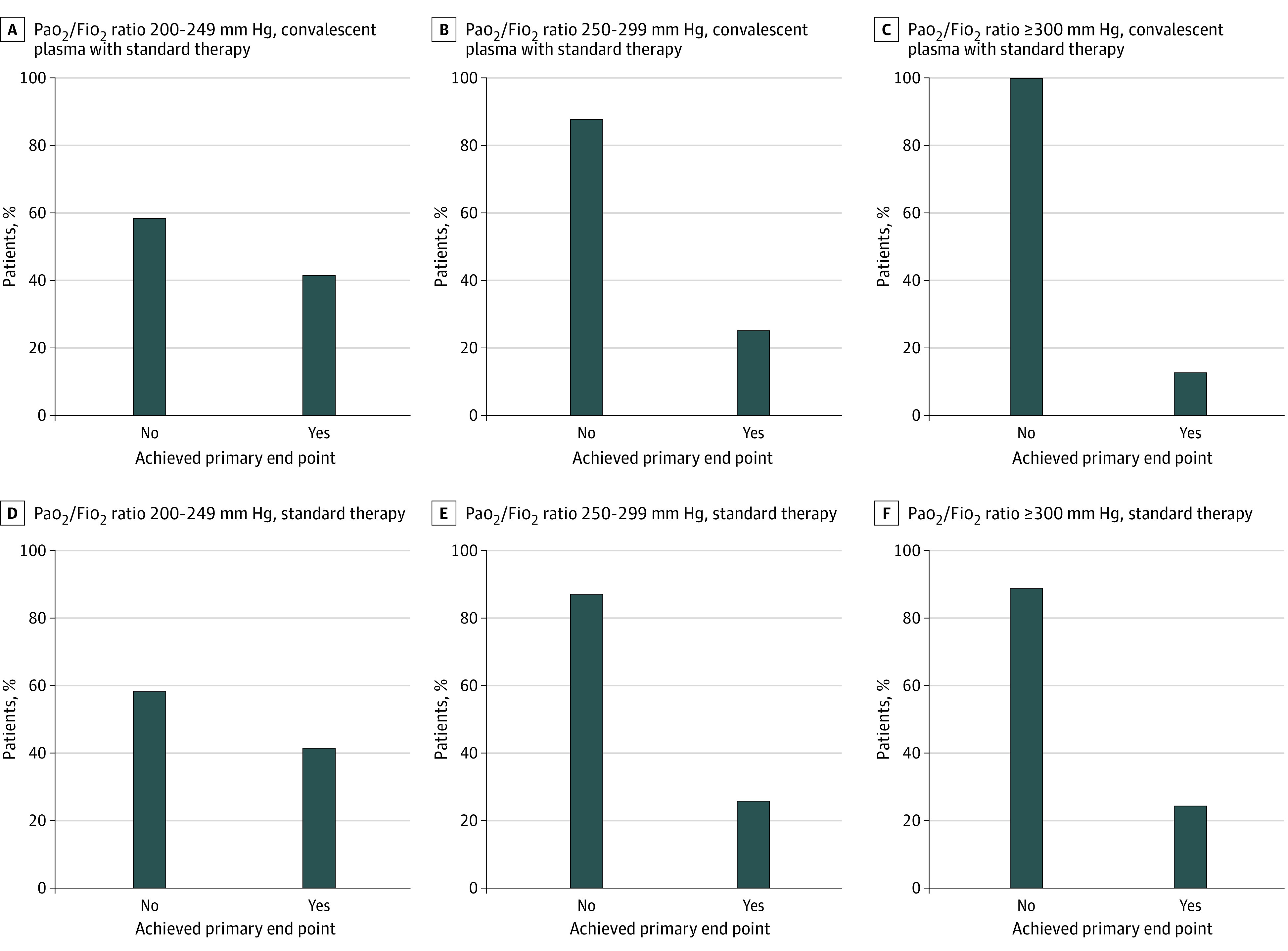
Primary End Point Distribution According to the Study Groups and Baseline Partial Pressure Of Oxygen–to–Fraction of Inspired Oxygen (Pao_2_/Fio_2_) Ratio Categories in the Modified Intention to Treat Population The primary end point was worsening respiratory failure or death within 30 days from randomization. Test for interaction: Pao_2_/Fio_2_ 250 to 299 mm Hg vs 200 to 249 mm Hg, *P* > .99; Pao_2_/Fio_2_ 300 mm Hg or greater vs 200 to 249 mm Hg, *P* = .06.

In the mITT population, the primary end point (a composite of worsening respiratory failure or death) occurred in 59 (45 worsening respiratory failure and 14 deaths) of 231 patients (25.5%) who received CP plus ST and in 67 (48 worsening respiratory failure and 19 deaths) of 239 patients (28.0%) who received ST alone (OR, 0.88; 95% CI, 0.59-1.33; *P* = .54). Data on the primary end point in the mITT population were missing for 3 patients: 1 in the CP plus ST group and 2 in the ST group. Results of sensitivity analyses for missing data confirmed results of primary analysis (eAppendix in Supplement 2). The analysis of primary end point in the PP and in the ITT population showed similar results (Table 2; eTable 4 and eTable 5 in Supplement 2). The subgroup analyses showed no statistically significant difference in the primary end point between the 2 treatment groups according to the prespecified subgroups (Figure 2; eTable 6 in Supplement 2). In patients with Pao_2_/Fio_2_ of 300 mm Hg or greater at baseline, the primary end point occurred less frequently in the group treated with CP plus ST (8 of 69 [11.6%]) vs those who received ST (16 of 73 [21.9%]), a nonstatistically significant difference (OR 0.47; 95% CI, 0.19-1.18). The distribution of the primary end point in the 3 groups of patients classified by basal Pao_2_/Fio_2_ ratio is reported on Figure 3. In the CP plus ST group, the primary end point occurred in 8 of 69 patients (11.6%) with a Pao_2_/Fio_2_ ratio of 300 mm Hg or greater at baseline and in 31 of 75 patients (41.3%) patients with a Pao_2_/Fio_2_ ratio from 200 to 249 mm Hg; conversely, in the ST group, the primary end point occurred in 16 of 75 patients (21.3%) with a Pao_2_/FiO_2_ ratio of at least 300 mm Hg at baseline and in 31 of 74 patients (41.9%) with a Pao_2_/FiO_2_ ratio from 200 to 249 mm Hg (*P* = .06). The analysis of primary composite end points for clinical sites is reported in eFigure 1 in Supplement 2. In 1 center, patients in the CP plus ST group had worse outcomes than those in the ST alone group.

**Table 2.** Primary End Point in the mITT and PP Populations^a^

Analysis population	No./total No. (%)		OR (95% CI)^b^	*P* value^c^
	Convalescent plasma plus standard therapy	Standard therapy		
mITT^d^	59/231 (25.5)	67/239 (28.0)	0.88 (0.59-1.33)	.54
PP	51/210 (24.3)	65/236 (27.5)	0.84 (0.55-1.29)	.43

Abbreviations: mITT, modified intention to treat; OR, odds ratio; PP, per protocol.

^a^ Primary end point was partial pressure of arterial oxygen–to–fraction of inspired oxygen ratio of less than 150 mm Hg or death at 30 days from randomization.

^b^ Crude estimates by univariate logistic regression models.

^c^ *P* value from χ^2^ test.

^d^ Data not available for 3 patients (1 in convalescent plasma plus standard therapy group; 2 in standard therapy group).

### Secondary End Points

The 30-day mortality rate was 6.1% (14 of 231) in the CP group and 7.9% (19 of 240) in the control group (*P* = .43). Mechanical ventilation or death, virological cure, and time from hospitalization to discharge were not significantly different in the 2 study groups (eTable 7 and eFigure 2 in Supplement 2). The rate of AEs was 5.0% (12 of 241) in the CP group and 1.6% (4 of 246) in the control group (*P* = .04). In patients receiving CP, 5 severe AEs requiring treatment interruption were documented: in all cases an acute worsening of respiratory failure was documented (in 2 cases, it was associated with fever and in 1 case with diffuse skin rash). The AEs had a low (2 events with worsening of respiratory failure without fever) and high (2 events with worsening of respiratory failure with fever and a skin rash) causality association with CP.

## Discussion

The TSUNAMI trial found that high-titer CP administered to hospitalized patients with moderate to severe COVID-19 pneumonia did not reduce the progression to severe respiratory failure or death within 30 days. These results were consistent across subgroups of age, sex, race, comorbidities, and use of concomitant therapy. Our data are in line with evidence from the literature. The PLACID study,^2^ which enrolled patients with inclusion criteria similar to the TSUNAMI trial, failed to demonstrate differences in the primary end point between CP and ST (19% vs 18%). Worsening of respiratory failure and mortality rates were slightly higher in our trial (25.5% and 28.0% for CP and ST, respectively) compared with the PLACID trial,^2^ probably due to the older study population of the TSUNAMI study (median age, 64 vs 52 years).

In the TSUNAMI trial, 30-day mortality was 6.1% for the CP group and 7.9% for the control group. Published^1,2,3,4,5,6,7,8,9^ and unpublished RCTs^10,11,12^ have failed to demonstrate any benefit of CP on mortality with the exception of the study by O’Donnell et al^14^ that found a significant reduction of 28-day mortality in patients receiving CP. In the RECOVERY trial,^4^ the mortality rate was 24.1% and 24.4% in the CP group and control group, respectively. These striking differences are difficult to explain and are probably multifactorial: difference in the population’s median age, difference in the risk factors for progression to severe COVID-19, time lapse between disease onset and CP transfusion, and differences in comprehensive patient management. It has been suggested that the benefits of CP may depend on the plasma NAb titer^9,13^ and that using CP with a low titer could explain negative results. To our knowledge, TSUNAMI was among the first and few RCTs using qualified CP with a NAb titer of at least 1:160 assessed with MNT. The serological IgG assays as surrogate markers of NAb titer in CP used in the other RCTs on CP is a reason for concern because the rate of correlation is highly variable.^25^ The presence of anti–SARS-CoV-2 IgG antibodies in recipients before receiving CP has also been cited as a possible reason for lack of effect of CP. Data from the RECOVERY trial^4^ demonstrates an improved outcome in CP recipients with no detectable anti–SARS-CoV-2 IgG antibodies at the time of transfusion (OR, 0.90; 95% CI, 0.82-0.97). It has been suggested that antibody-based therapies are likely to be most effective in the early stages of COVID-19, when viral replication dominates. In our trial, the median time from the onset of COVID-19 to CP administration was 7.7 days. In the trial by Libster and coworkers,^15^ high-titer CP administered within 72 hours from the onset of mild COVID-19 reduced the progression of the disease in older adults (severe respiratory failure occurred in 16% of patients treated with CP vs 31% of those who received placebo; *P* = .03).

In our study, AEs occurred more frequently in the CP group, although published reports on very large patient cohorts documented a good CP safety profile.^26^ Of note, we did not observe any thrombotic events in the group of patients receiving CP. The hypothesized prothrombotic effect of plasma and the risk in the context of a thrombophilic disorder like COVID-19^27^ has not been formally demonstrated in vivo.

### Limitations

Our study has several limitations. The sample size of the TSUNAMI trial was inadequate for the analysis of subgroups, such as patients with mild pneumonia, early disease onset (<72 hours), and negative serology. Therefore, further studies are needed to evaluate the role of high-titer CP therapy in these groups of patients.

Quite surprisingly, in 1 of the main centers, patients treated with CP had a worse outcome compared with the control group. A careful investigation was performed but neither deviations from the study protocol, nor differences in the study population were identified: we were unable to document any difference in the severity of population treated, NAb concentration in the transfused CP plasma, baseline anti-S IgG levels, or time from randomization to CP infusion.

## Conclusions

This randomized clinical trial enrolled patients with moderate to severe COVID-19 pneumonia. High-titer CP did not reduce the progression to respiratory failure or death within 30 days among these patients vs those receiving ST.

## Notes

Supplement 1.
Trial Protocol and Statistical Analysis Plan

Supplement 2. **eAppendix. **Statistical Analysis **eTable 1. **Characteristics of the Patients at the Baseline in the Randomized Population **eTable 2. **Convalescent Plasma Treatment During Trial, mITT Population **eTable 3. **Oxygen Supplementation and Treatment During Trial (Day 1 to Day 30 Post Randomization) in the mITT Population **eTable 4. **Characteristics of the Patients at the Baseline in the ITT Population **eTable 5. **Primary End Point in the ITT Population **eTable 6. **Primary End Point in the mITT Population According to Lag From Onset of Symptoms to Convalescent Plasma (CP) Infusion **eTable 7. **Secondary End Points in the mITT Population **eFigure 1. **Primary End Point Outcome According to Trial Group and Clinical Sites in the mITT Population **eFigure 2. **Survival Curves at 30 Days of Secondary Outcomes in the mITT Population
eReferences.

Supplement 3.
Nonauthor Collaborators

Supplement 4.
Data Sharing Statement
