# Errors in Results, Figure 1, Table 1, and Article Information

**DOI:** 10.1001/jamanetworkopen.2021.44236

**Published:** 2021-12-21

In the Original Investigation titled “Effect of High-Titer Convalescent Plasma on Progression to Severe Respiratory Failure or Death in Hospitalized Patients With COVID-19 Pneumonia: A Randomized Clinical Trial,”^1^ published November 29, 2021, there were errors in the Results section, Figure 1, Table 1, and the Article Information section. In the subsection “Primary End Point” in the Results section, the number of patients in the standard therapy group with a Pao_2_/Fao_2_ of at least 300 mm Hg who experienced the primary end point was 16 of 73 (21.9%). In Figure 1, the number of patients in the standard therapy group after 3 participants withdrew informed consent was 243. In Table 1, data were missing for the Sequential Organ Failure Assessment score for 106 patients. In the Article Information section, Dr Spila should have been included in the list of members of the TsuStat group. This article has been corrected.^1^
