# Error in Article Information

**DOI:** 10.1001/jamanetworkopen.2021.46944

**Published:** 2022-01-18

In the Original Investigation titled “Effect of High-Titer Convalescent Plasma on Progression to Severe Respiratory Failure or Death in Hospitalized Patients With COVID-19 Pneumonia: A Randomized Clinical Trial,”^1^ published on November 29, 2021, the authors omitted the following disclosures: Dr Menichetti reported serving as the principal investigator for an AstraZeneca–sponsored trial and a Toscana Life Science-sponsored trial evaluating monoclonal antibodies for SARS-CoV-2 (for which no personal fees were received), and receiving speaker honoraria or advisory board or support for meetings from Angelini, Menarini, Correvio, MSD, Pfizer, Astellas, Gilead, BMS, Janssen, ViiV, BioMerieux, Biotest, Becton-Dickinson, Pfizer, Shionogi, Roche, GSK, Advanz Pharma, and ThermoFisher in the last 3 years (outside the submitted work); Dr Bartoloni reported receiving study grants from MSD, ViiV Healthcare, and Nordic Pharma and receiving fees for presentations at local congresses or expert meeting from Pfizer and MSD in the last 3 years (outside the submitted work); Dr Marchetti reported receiving grants for lectures, advisory board, or conferences by Gilead, ViiV, and Janssen in the last 3 years (outside the submitted work); Dr d’Arminio Monforte reported receiving grants for lectures, advisory board, or conferences by Gilead, ViiV, MSD, Angelini, and Janssen in the last 3 years (outside the submitted work); Dr Saracino reported receiving grants for research and/or educational purposes from Gilead, ViiV, MSD, Abbvie, Janssen, Shionogi, and Pfizer in the last 3 years (outside the submitted work); Dr Castagna reported receiving personal fees from ViiV, MSD, Gilead, Janssen, and Theratecnologies in the last 3 years (outside the submitted work); and Dr Falcone reported receiving grants and speaker honoraria from Angelini, Shionogi, MSD, Pfizer, Gilead, Menarini, and Nordic Pharma in the last 3 years (outside the submitted work). The corresponding author has explained this omission in a Comment^2^ and the article has been corrected.^1^ This article was previously corrected on December 21, 2021, to correct errors in the Results section, Figure 1, Table 1, and Article Information.^3^
