# Supplementary material for: Effect of High-Titer Convalescent Plasma on Progression to Severe Respiratory Failure or Death in Hospitalized Patients With COVID-19 Pneumonia: A Randomized Clinical Trial
Source: JAMA Netw Open. 2021 Nov 29;4(11):e2136246. doi: 10.1001/jamanetworkopen.2021.36246 (PMC8630572; doi:10.1001/jamanetworkopen.2021.36246)
Supplement: Supplement 1. — Trial Protocol and Statistical Analysis Plan [file jamanetwopen-e2136246-s001.pdf]

Randomized clinical trial Protocol

TranSfUsion of coNvalescent plAsma for the early treatment of pneuMonIa due to SARSCoV2 (TSUNAMI Study): a multicenter open label randomized control trial

TSUNAMI Study

Version 5.0

Date 18/05/2021

Sponsor: Istituto Superiore di Sanità
Collaborators: Agenzia Italiana del Farmaco
Gruppo Italiano Malattie EMatologiche dell'Adulto

Principal Investigator: Prof. Francesco Menichetti
Infectious Disease Unit, Azienda Ospedaliera Universitaria Pisana
Department of Clinical and Experimental Medicine, Univerisity of Pisa
Pisa, Italy

BACKGROUND

Since the end of 2019, the SARS-CoV-2 spread from the province of Hubei, Wuhan, to all countries in the world. The SARS-CoV-2 is the causative pathogen of a syndrome called new coronavirus 2019 (COVID-2019, Coronavirus disease 2019) whose clinical picture includes a spectrum disease ranging from a simple flu syndrome to pneumonia with severe insufficiency respiratory often fatal. The virus has a specific tropism for the epithelium pulmonary. Already at the beginning of 2020 the first cases were registered also on our country and in February 2020 the first case of transmission was reported in Italy. There is currently no specific treatment for SARS-CoV-2. Passive immunization for the prevention and treatment of human infectious diseases and the concept correlated with artificially acquired passive immunity dates back to the initial experience of use for infection from hepatitis B virus, avian, and more recently from Ebola virus. The ability to get immediately immunization against infectious agents by administering against specific the pathogen contained in the plasma obtained from the recovered / convalescent subjects has shown a possible efficacy in patients lacking therapeutic alternatives. As happened for other previous viral epidemics, such as Ebola, MERS-CoV (Middle East respiratory syndrome coronavirus, MERS-CoV), H1N1pdm09 (2009 influenza A H1N1 pandemic), the use of plasma from convalescent subjects can have a therapeutic role, because it is feasible and inexpensive. Clinical data on convalescent plasma are limited. A meta-analysis was conducted in 2015 (J Infect Dis 2015; 211: 80-90) to underestimate the effectiveness clinic of the administration of plasma or serum for the treatment of acute respiratory infections of etiology viral in order to be able to clinically manage MERS-CoV infection. Despite the studies analyzed were considered to be of low quality, with no control groups and with moderate risk or elevator bias, the authors concluded that the administration of plasma or serum from immunized patients could be applied in a safe way, recording a reduction in hospital stay and above all less mortality. One possible explanation for the efficacy of convalescent plasma is the reduction of the viraemia. Generally, the viral load peaks in the former week of infection and the patient develops a primary immune response within days 10–14, followed by the clearance of the virus. For this reason, it may be more effective to administer convalescent plasma in the initial stage of the disease. Very recently, the first evidence has emerged about the efficacy of plasma therapy in patients with COVID-19. A first study published

in JAMA on March 27, 2020 documented a positive response to the infusion of convalescent plasma in 5 patients admitted to the Intensive Care Unit in China (JAMA 2020; March 27). A further Chinese study in 10 patients confirmed the potential of plasma therapy in improve the prognosis of COVID-19 patients (Proc Natl Acad Sci USA 2020; Apr 6).

The availability of plasma donors allows the creation of a specific immunity acquired against the infectious agent against the local viral strain, which could change over time compared to the wild type strain. The possibility of collecting plasma by plasmapheresis procedure making it immediately available to the patients represents at this moment a further therapeutic possibility. The early use of plasma could play an important role in reducing the risk of ARDS and ICU transfer. The hypothesis of the study is that the early use of convalescent plasma can reduced the need of mechanical ventilation and improve the survival and length of stay of these patients. Furthermore, we aim to explore the time to negative nasopharyngeal swab in patients treated with plasma.

To date there are no studies in the literature that demonstrate its feasibility and effectiveness in the field of the worldwide SARS-CoV-2 pandemic.

OBJECTIVES

Primary objective

The primary objective of the study is to evaluate the effectiveness of the early administration of plasma from convalescent donors from COVID-19. The effectiveness will be evaluated as a composite of need of mechanical ventilation or death. The need for invasive mechanical ventilation will be defined as reduction of PiO2/FiO2 <150.

Secondary objective

Secondary objective is to evaluate the clinical outcome of patients treated in terms of:

- 30-day mortality
- time (days) to mechanical ventilation
- time (days) to virological recovery (negativization of 2 consecutive nasopharyngeal swabs)
- duration of hospitalization
- safety objectives in relation to any adverse events that may occur

Outcome Measures

Primary Outcome Measures :

Number of patients who meet invasive mechanical ventilation or death [Time Frame: at 30 days]
Number of patients who meet invasive mechanical ventilation defined as PaO2/FiO2 <150 or death

Secondary Outcome Measures :
Mortality rates at 30 days
Time to invasive mechanical ventilation or death [Time Frame: 30 days]
Days from randomization to invasive mechanical ventilation or death
Time to virologic recover [Time Frame: 30 days]
Days from randomization to virologic recover (defined as 2 consecutive negative nasopharynx tests)
Hospitalization time [Time Frame: 30 days]
Adverse events [Time Frame: 30 days]

Study design

Randomized controlled multicentre, national, open label, no-profit study

Recruitment of convalescent donors

Convalescent donors will be recruited from the participating Transfusion Services (TS), which will identify all the candidates to be selected for donation, with particular reference to donation in apheresis. All donations through plasmapheresis will be performed at the individual TSs by adopting the "Operating protocol for the selection of convalescent patient-donors with a virologically documented diagnosis of COVID-19, for the biological qualification of the plasma from apheresis possibly produced as well as for subsequent correlates procedures for the reduction of pathogens and controlled storage ", drawn up by the National Blood Center (appendix 1).

Duration of the study: 6 months

STUDY POPULATION

The study will be conducted in hospital setting and include patients selected on the basis of the criteria described below.

ELIGIBILITY CRITERIA

donor inclusion criteria

- age 18-65 years
- confirmed diagnosis of previous COVID-19
- presence of two negative nasopharyngeal swabs (if the donor is a convalescent cured with a previous positive swab) at least 14 days after the second negative nasopharyngeal swab.
- subject with no previous virological diagnosis but with a positive serological test for IgG and the presence of a negative nasopharyngeal swab at least 14 days after any previous symptoms.
- presence of adequate levels (1:160) of neutralizing antibodies to SARS-COV-2
- informed consent signature

Donor exclusion criteria
- age <18 years or> 65 years
- other criteria included in the "Operational protocol for the selection of diagnosed convalescent patient-donors virologically documented of COVID-19, for the biological qualification of plasma from apheresis possibly produced as well as for the subsequent related procedures for the reduction of pathogens and controlled storage ", drawn up by the National Blood Center and attached to this study protocol.

Inclusion Criteria for Patients:
- Age ≥ 18 years old
- adult patients with positive RT-PCR test for SARS-CoV2 (nasal swabs or lower respiratory tract sample), diagnosed with pneumonia (<= 10 days) according to the following definitions:
  - Suggestive radiological imaging (CT, RX, ultrasound);
  - Respiratory failure not fully explained by heart failure or fluid overload;
  - PaO2/FiO2 200-350 mmHg;
- Signed informed consent

Exclusion Criteria for Patients
- need of non invasive or invasive mechanical ventilation at the time of randomization;
- PaO2/FiO2 <200;
- patients with hypersensitivity or allergic reaction to blood products or immunoglobulins;
- patients who expressly refuse to adhere the clinical study;
- use of IL-6 Receptor inhibitors, IL-1 inhibitors, JAK inhibitors, TNF inhibitors;

- patients participating to other clinical trials

Withdrawal Criteria

Withdrawal of consent

INTERVENTIONS

Plasma collection from convalescent donor

The donation of plasma, by plasmapheresis (volume from 600 to 700 mL, excluding the anticoagulant), must be carried out in compliance with current transfusion regulations. Furthermore, the indications referred to in the Operational Protocol for the selection of convalescent patients-donors with diagnosis virologically documented of COVID-19, for the biological qualification of plasma from apheresis possibly produced as well as for the subsequent related procedures for the reduction of pathogens and controlled storage, drawn up by the National Blood Center and attached to this study protocol.

The plasma donation should follow the following steps according to the current transfusion legislation:

1) Acceptance of the convalescent donor according to current transfusion regulations

The registration of the donor's personal data is carried out by the staff of the participating TSs through IT management systems with procedure compliant with current blood transfusion regulations, prior acquisition of consent to the processing of personal data by the donor / convalescent. For each convalescent patient-donor registered, the IT management system generates a code identification data univocally associated with the donation. The donation is identified with a code unique, compliant with transfusion regulations, reproduced on the identification label.

2) Selection of the convalescent donor

The selection of donors is carried out according to the indications of the Operational Protocol drawn up by the National Blood Center and attached to this study protocol.

3) Collection procedure using cell separator

The convalescent donor, duly informed about the apheresis procedure, releases the informed consent to donation. The collection of hyperimmune plasma will be performed through use of a cell separator supplied with the TS. The donation will take place at times other than those in which they access

usually donors, to avoid contact. Nursing assistance will be guaranteed on an ongoing basis.

4) Biological qualification of hyperimmune plasma

See Operating Protocol drawn up by the National Blood Center and attached to this protocol of study.

5) Procedure of pathogen reduction, freezing and preservation of hyperimmune plasma

Each plasmapheresis will be aliquoted in sub-units of volume between 200 and 300 mL. Pathogen reduction, freezing and storage of hyperimmune plasma will be carried out, in compliance with the indications of the transfusion regulations, according to the validated operating procedures in participating TSs.

Each plasmapheresis will be stored, in accordance with the national regulations, separately from other units intended for clinical use or industrial fractionation. If requested by the user center, the hyperimmune plasma units can be stored at + 4 ° C (± 2 ° C) and administered within 24 hours of collection.

6) Distribution of the blood component to the user department

The assignment to the patient-recipient of fresh frozen plasma (according to study inclusion criteria and in accordance with the randomization procedure in use for all centers participating in the study) and the subsequent transfusion of the aforementioned blood component will take place in full compliance with current legislation provisions on transfusion safety, traceability and haemovigilance. Assignment to the patient (in accordance with the randomization procedure in use for all centers study participants) and delivery to the user department will be tracked through the systems IT management of the participating TSs. The exact dose of plasma to be infused (in the range of 200-300 mL for a maximum of 3 times over the period 5 days) will be decided on the basis of the characteristics of each patient. In order to monitor the viraemia and the immune response, 2 peripheral blood samples will be collected without anticoagulant and 2 in EDTA immediately before and 24 hours after the plasma infusion hyperimmune.

Evaluation of the potential benefit / risk ratio for the population

The potential risks for the study population are the followings:

- for donors: possible undesirable reactions, immediate or late, related to the donation of plasma by apheresis.
- for recipients: possible side effects, of mild, moderate or severe severity, attributable to the transfusion of plasma.

Withdrawal of subjects and modifications of the intervention

The withdrawal of the subjects will be possible in case of withdrawal of the informed consent.

The intervention is suspended in case of correlated adverse events

Assignment to the experimental group:

Patients who meet the inclusion criteria will be enrolled by the attending clinician and, through the GIMEMA platform, randomized in a 1: 1 ratio to one of the following groups:

a) administration of hyperimmune plasma in addition to standard therapy. The exact dose of plasma from infuse will be decided on the basis of the clinical condition of each patient. A range of 200-300 ml for a maximum of 3 times within 5 days.

b) standard therapy (see below)

Standard therapy

All patients, regardless of the assignment arm, will continue to receive standard therapy. Standard therapy will be carried out in accordance with the indications of the AIFA data sheets regarding both the choice of drugs, dosages and durations of treatment. The concomitant use of IL-6 receptor inhibitors, IL-1 inhibitors, JAK inhibitors, TNF inhibitors is not allowed from the randomization.

Rescue Therapy

Patients will be monitored according to normal clinical practice. In case of worsening of clinical conditions, the Investigator can decide whether to introduce other drugs as rescue therapy.

Early termination of the study

The promoter may interrupt the study at any time and promptly notify them investigators and ethics committees. Patients will be seen as soon as possible and will continue to be followed up according to normal clinical practice.

Definition of study conclusion

The patient will be followed for 30 days after plasma administration (30 day follow-up) in order to evaluate the possible occurrence of adverse events. In general, the conclusion of the study will coincide with the end of follow-up of the last patient enrolled.

STUDY PLAN

Study timeline

Enrollment period: May 2020 - September 2020

Sample size

Considering the daily provided and published data, mechanical ventilation in the absence of treatment is needed in 30% of patients (P0 = 0.30). The study size was calculated to highlight one 40% reduction in the primary endpoint (delta = 0.12; P1 = 0.18), a statistical power of 80% and an error alpha of 5%, with statistical test for comparing two-tailed proportions, and a 1: 1 randomization ratio. By adopting a sequential design with 2 interim analyzes, it is planned to conduct the first interim analysis with 120 patients enrolled, the second interim analysis with 238 patients enrolled and the final analysis with 474 patients. Details on how to calculate the sample size can be found in the Plan section.

THE CALCULATIONS WERE CARRIED OUT USING THE RPACK SOFTWARE (CONFIRMATORY ADAPTIVE CLINICAL TRIAL DESIGN AND ANALYSIS, R PACKAGE).

DATA MANAGEMENT

Data collection

Data collection will be carried out continuously (daily) for the entire duration of the treatment through a database developed in REDCap (a tool for collecting data from clinical study). The data will be recorded via electronic Case Report Forms (eCRF) e inserted in the REDCap system. .

Data management

The GIMEMA Foundation will be in charge of the data management of the study. (see Appendix: Management clinical study). All data will be pseudonymized. The processing and storage of data will take place in compliance with the law on the protection of personal data (Legislative Decree 30/6/2003 n. 196, Lines guide for the processing of personal data in the context of clinical trials of medicines - 24 July 2008 - G.U. n. 190 of 14 August 2008 and the EU Regulation 2016/679, known as GDPR (General Data Protection Regulation).

The investigators who care for the patient are responsible for the information of the patient and for obtaining the informed consent. Consent may be oral if it is not possible to express written consent. If the subject is unable to provide informed consent and an authorized representative is not available without delays which, in the Investigator's judgment, would compromise the potential life-saving effect of the treatment, this can be administered without consent. Consent to remain in research must be acquired as soon as the patient's condition allows. The same procedure applies to patient information and providing consent to data processing personal according to the European Regulation n. 679/2016 on the protection of personal data, the code of protection of personal data (legislative decree 196/03) and subsequent amendments and additions, as well as to the provisions, guidelines and general authorizations of the national supervisor for the protection of personal data.

Data retention

The pseudonymised data will be stored on the REDCap platform for a period not exceeding that necessary for the purposes of the study for which they were collected (and in any case no later than 10 years from conclusion of the study), always in accordance with the mandatory retention periods defined by law. Any personal data will be deleted after the applicable retention period expires. In any case, the participating subjects have the right to request, at any time, the deletion of data, in compliance with the GDPR and the applicable data protection law. In compliance with the privacy legislation, the data will be sent to the Promoter and Coordinator Center of the study that will use them for data processing and analysis.

Administrative aspects

The study is not for profit. The stipulation of a suitable insurance policy is required.

STATISTICAL PLAN

The primary analysis will be conducted in the Intention To Treat (ITT) population. In the primary analysis, the percentages of subjects who will need assisted ventilation in the two experimental groups will be compared with the 2-tailed chi-square test. The proportions observed in the two groups will be presented with relative 95% confidence interval (95% CI). Categorical variables will be described as scores and percentages, quantitative ones with mean and standard deviation if normally distributed or median e interquartile range. The time course of the parameters indicating the viral load and the immune response they will be studied with models for repeated measures over time. The proportion of grade ≥3 adverse events will be described, together with 95% CI. An ad hoc SAP will be prepared which will include all the procedures for the treatment of any missing data and any sensitivity analyzes.

Calculation of the sample size

Considering the daily provided and published data, mechanical ventilation in the absence of treatment is needed in 30% of patients (P0 = 0.30). The study size was calculated to highlight one 40% reduction in the primary endpoint (delta = 0.12; P1 = 0.18). A statistical power of 80% and an alpha error of 5% was considered, with statistical test for comparison between two-tailed conducted proportions and a 1: 1 randomization ratio. Based on these assumptions, adopting a sequential design with 2 interim analyzes, the Pocock method for checking the family-wise type I error, and using information rates of 0.25 and 0.50 for the former and the second interim analysis respectively, it is planned to conduct the first interim analysis with 120 patients enrolled, the second interim analysis with 238 enrolled patients and a possible final analysis with 474 patients. With these specifications it will be possible to highlight a Minimum at the first interim analysis Detectable Effect (MDE) of about 60%). Based on the enrollment rate found, it will be possible decide to unbalance the randomization ratio in favor of the plasma group (2: 1). If so, yes will proceed with the recalculation of the sample size.
Calculations were conducted using RPACK (Confirmatory Adaptive Clinical Trial Design and Analysis, R package).

Randomization

Being an open, parallel, multicentre study, the assignment of a patient to one of the two arms it will take place through a centralized block randomization procedure with a stratification factor for clinical center. The randomization list will be generated by the coordinating center via procedure electronic

validated by STATA ® software After verifying the presence of the inclusion / exclusion criteria the investigator will assign a sequential randomization code. Patient randomization will be edited by GIMEMA on the basis of the randomization list sent by the coordinating center.

Pairing procedure

In the event of difficulty in recruiting, even following the possible introduction of the rate of randomization of 2: 1, or the non-feasibility of the randomized study, the advisability of follow a retrospective cohort approach (albeit with less solid foundations) by identifying an appropriate group of controls, following an individual pairing to the patients enrolled in the experimental arm. Each patient belonging to the experimental arm will be paired (1: 1) with another patient of a cohort retrospective of inpatients in the same center and in a near period of time. The matching will be performed considering the center, age, sex, hospitalization period, severity, as matching variables, and possible concomitant therapies among those provided as standard of care. This analysis will be pre-specified with all the statistical details in an amendment to the protocol before the conclusion of enrollment e access to data by defining the pairing criteria and endpoints (primary and secondary) that will be possible compare. As a further supportive analysis (limited to the mortality endpoint), a comparison with survival curves of the hospitalized population of the ITA-COVID network.

Following a counterfactual approach, each patient treated in the experimental arm will be assigned one expected mortality (at fixed time point) equal to that (for the same sex and age characteristics) of the ITACOVID cohort thus defining the expected mortality of a hypothetical control group with the same characteristics by age and sex of randomized patients. A comparison will then be made between the expected mortality and observed.

SAFETY MANAGEMENT

Adverse event reporting

The investigator will enter in the REDCap platform any adverse event or laboratory results no later than 24 hours after the event. If necessary, the Promoting Center may request the investigator to follow up on the event. It will be created a Data and Safety Monitoring Board (DSMB) which will periodically review adverse events to reassess the risk / benefit ratio of the treatment.

Reporting of adverse reactions to plasma

The recording of each adverse reaction is foreseen, in accordance with the EMA GVPs (https://www.ema.europa.eu/en/humanregulatory/postauthorisation/pharmacovigilance/goodpharmacovigilance-practices), both in plasma donors and recipients. Such reactions will be recorded using the National Information System of Transfusion Services (SISTRA) relating to haemovigilance.

Reporting of adverse drug reactions

In case a suspected adverse reaction to drugs used for standard therapy is observed, in accordance with Regulation (EU) 536/2014, the report must be registered in the Clinical Trial module of Eudravigilance.

ETHICAL CONSIDERATIONS

The study will be conducted in accordance with the ethical principles enshrined in the Helsinki Declaration in its latest revision. Participation in the study will be subject to obtaining free and informed consent (attached) and the rights enshrined in the law regarding the protection of personal data will be safeguarded (General EU regulation on the protection of personal data 679/2016; Legislative Decree 30/6/2003 n. 196 and subsequent amendments; Requirements relating to the processing of personal data carried out for the purposes of scientific research by the Authority for the protection of personal data - aut. gen. n. 9/2016) of the subjects included in the study.

Acquisition of informed consent

Informed consent will be acquired from all patients included in the study, including incapacitated or in emergency cases, by means of an approved informed consent form. Consent can be oral if not it is possible to express written consent. If the subject is unable to provide informed consent and a authorized representative is not available without delay which, in the opinion of the Investigator, would compromise the potential life-saving effect of the treatment, this can be administered without consent. Consent to stay in research must be acquired as soon as the patient's condition is will allow.

Confidentiality

All information on personal data of eligible subjects before, during and after the trial will be processed confidentially, in accordance with current legislation.

Conflict of interest

No conflict of interest

Appendix 1: Calculation of the sample size

Parameters used in the calculation of the sample size:

- H0: P0 = 0.3
- H1: P1 = 0.18 (delta = 0.12)
- alpha = 5%
- Power = 80%
- two-tailed test
- Sequential design with 2 interim analyzes
- Pocock's method for checking the family-wise type I error
- Information rates of 0.25 and 0.50 for the first and second interim analysis respectively

Summary Table

```
Sample size calculation for a binary endpoint

Sequential analysis with a maximum of 3 looks (group sequential design).
The sample size was calculated for a two-sample test for rates (two-sided),
treatment rate pi (1) = 0.18, control rate pi (2) = 0.3, allocation ratio = 1, and power 80%.

Stage                                        1      2      3
Information rate                           25%    50%   100%
Efficacy boundary (z-value scale)        2.312  2.312  2.312
Number of subjects                         119    237    474
Cumulative alpha spent                  0.0208 0.0358 0.0500
Cumulative power                        0.2182 0.4755 0.8000
Two-sided local significance level      0.0208 0.0208 0.0208
Lower efficacy boundary (t)             -0.174 -0.128 -0.092
Upper efficacy boundary (t)             -0.174 -0.128 -0.092
Exit probability for efficacy (under H0) 0.0208 0.0151
Exit probability for efficacy (under H1) 0.2182 0.2573

Legend:
  (t): approximate treatment effect scale
```

Minimum Detectable Effect (MOE)

Anal isi	***Int erim 1***	***Inte rim 2***	***Fi nale***
MDE	***-0, 174***	***-0 , 128***	***-0,092***
Diffe renza % ***(vs 30 %, gruppo di controllo)***	***58 %***	***43 %***	***30%***

R commo nd

```
design <- getDesignGroupSequential(typeOfDesign = "P",  informationRates = c(0.25,  0.5,1), alpha =  0.05,  sided  =
2)
print(design)
getDesignCharacteristics(design)
design P /an < - getSampleSizeRates{design, pil "' 0.18, pi2 "'0.3)
summary(designP/an)
print(designP/an)
```

Plot 1: Rate Diffe rence

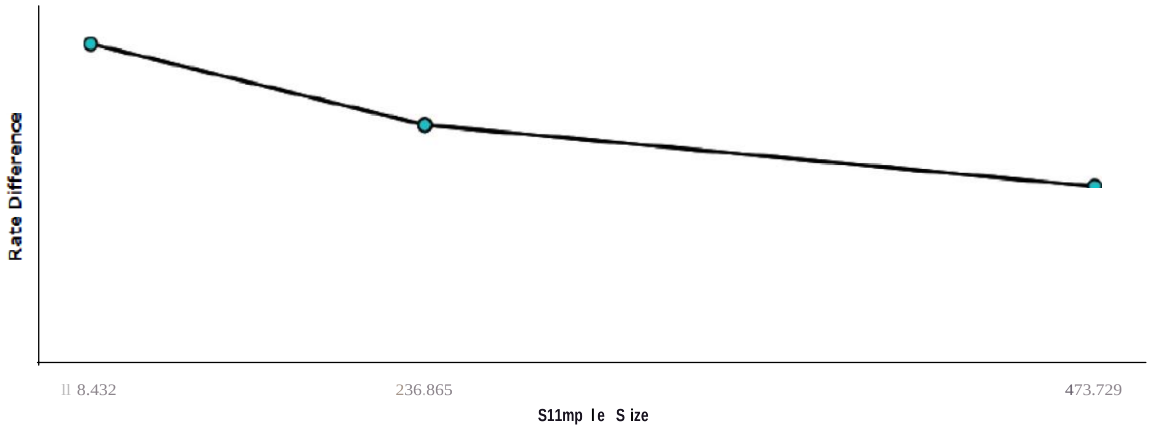

Plot 2: Cumulative alpha spending

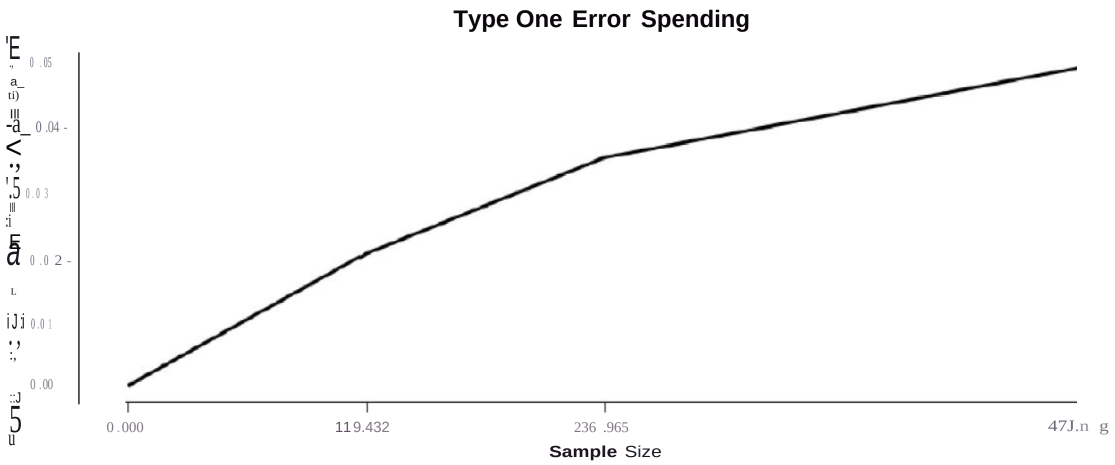

Randomized clinical trial Protocol

TranSfUsion of coNvalescent plAsma for the early treatment of pneuMonIa due to SARSCoV2 (TSUNAMI Study): a multicenter open label randomized control trial

TSUNAMI Study

Version 7.0

Date 21/01/2021

Sponsor: Istituto Superiore di Sanità (ISS)

Co-Sponsor: Agenzia Italiana del Farmaco (AIFA)

Collaborator: Gruppo Italiano Malattie EMatologiche dell'Adulto

Principal Investigator: Prof. Francesco Menichetti

Infectious Disease Unit, Azienda Ospedaliera Universitaria Pisana

Department of Clinical and Experimental Medicine, University of Pisa

Pisa, Italy

Co-Principal Investigator: dr Cesare Perotti

IRCCS, Foundation San Matteo General Hospital, Pavia, Italy

Note: The present clinical protocol Version 7.0 is the last one approved by the unique EC INMI 'L. Spallanzani'. The original Version 5.0 underwent four amendments.

SYNOPSIS

Title

TranSfUsion of coNvalescent plAsma for the early treatment of pneuMonIa due to SARS-CoV2 (TSUNAMI Study): a multicenter open label randomized control trial (TSUNAMI study)

Primary objective of the study is to evaluate the efficacy of plasma obtained from convalescent COVID-19 donors and early administered to COVID-19 positive patients. The efficacy will be evaluated as the decrease in the percentage of patients who need mechanical ventilation (defined as first occurrence of a P/F ratio value <150 within 30 days from the randomization of the patient), or death within 30 days.

Study design

Randomized controlled multicenter, national, open label, no-profit clinical trial.

Convalescent donors

Convalescent donors will be recruited by the participating Transfusion Services (TSs). All blood donations will be performed at the specific TS following the protocol established by the National Blood Center (Annex 1). To be eligibile for transfusion, plasma must have a titer of neutralization antibodies ≥1:160.

Study population

Main inclusion criteria for donors.

- age 18-65 years
- confirmed diagnosis of previous COVID-19 disease
- Recovery from COVID-19 infection according to clinic and laboratory molecular tests criteria specified by the guidelines released by the Italian Ministry of Health on 12 October 2020: negative nasopharyngeal swab after at least 10 days from symptoms onset (not considering anosmia and ageusia/dysgeusia) and at least 3 days without symptoms.

- in case of previously hospitalized donors, it is recommended to perform the apheresis after at least 28 days from the documented recovery.

- presence of adequate levels (≥1:160) of neutralizing antibodies against SARS-COV-2

- informed consent signature

Main inclusion criteria for recipients

- age≥18 years
- patients positive for SARS-CoV-2 diagnosed by RT-PCR performed on nasopharyngeal swabs or on deep breathing samples, pneumonia diagnosis since ≤10 days based on the following definitions:
  - Suggestive radiologic imaging (TC, RX, Ecography)
  - Respiratory failure not completely explainable by cardiac failure or fluid overload
  - PaO2/FiO2 200-350 mmHG

Main recipient exclusion criteria (at least one of the following)

- Invasive or non-invasive mechanical ventilation (CPAP/NIV) at randomization
- PaO2/FiO2 <200

Assignment to the experimental group

The patients will be randomized 1:1 to the following arms:

a) Plasma hyperimmune therapy in addition to standard therapy. The exact amount of plasma to be transfused will be established based on the clinical status of each patient. It is recommended a range of 200-300 ml for maximum 3 times over 5 days.
b) Standard therapy (in accordance with the AIFA guidelines with regard to the choice of drugs, their dosage and the treatment duration).

Patient assignment of either of the two arms will be performed by a centralized randomization procedure with a stratification factor for each clinical site. The randomization list will be produced by the coordinating center through a validated electronic procedure.

Rescue therapy

The patients will be monitored according to the regular clinical practice. In case of worsening of the clinical conditions, the Investigator can decide to use other drugs as rescue therapy.

End of study

Each patient will be followed for 30 days from the randomization (follow-up = 30 days) in order to evaluate treatment efficacy and safety. End of study will correspond to the end of the follow-up of the last enrolled patient (last patient last visit).

Primary endpoint

In agreement with the primary objective the efficacy will be evaluated as a decrease in the percentage of patients who are considered in need of mechanical ventilation (defined as first occurrence of a P/F ratio value<150 within 30 days from the patient randomization date), or death within 30 days.

Patient number

474 patients fulfilling the inclusion and exclusion criteria described in the protocol will be enrolled

Expected study duration

12 months from the enrollment of the first patient.

Adverse events reporting

The investigator will report all the adverse events (including laboratory abnormalities, symptoms or diseases concomitant to the treatment, but not necessarily related to it) in the REDCap platform. All adverse reactions occurring both in plasma donors and recipients, will be notified through the National Informative System of the Transfusion Services (SISTRA).

Data Safety and Monitoring Board

An independent committee (Data Safety and Monitoring Board, DSMB) is in charge to regularly evaluate the collected data with regard to patient safety, study conduction and progress, and

efficacy of the treatment. In addition, the committee may provide recommendations regarding trial extension, possible modifications or premature conclusion.

BACKGROUND

Since the end of 2019, the SARS-CoV-2 spread from the province of Hubei, Wuhan, all over the world. The SARS-CoV-2 is the causative pathogen of a syndrome called new coronavirus 2019 (COVID-2019, Coronavirus disease 2019) whose clinical picture includes a spectrum disease ranging from a simple flu syndrome to pneumonia with severe respiratory insufficiency, often fatal. The virus has a specific tropism for the pulmonary epithelium. The first SARS-CoV-2 cases were registered in our country at the beginning of 2020 and the first case of disease transmission was reported in Italy on February 2020. No specific treatment for SARS-CoV-2 is currently available. Passive immunization for the prevention and treatment of human infectious diseases and the concept of artificially acquired passive immunity were firstly experienced in the hepatitis B virus infection, avian infection, and more recently, in the Ebola virus infection. The capability to obtain a prompt immunization against infectious agents through the administration of plasma obtained from the recovered/convalescent subjects and containing specific antibodies against those pathogens has previously demonstrated a potential efficacy in patients lacking therapeutic options. As happened for ~~other~~ previous viral epidemics, such as Ebola, MERS-CoV (Middle East respiratory syndrome coronavirus, MERS-CoV), H1N1pdm09 (2009 influenza A H1N1 pandemic), the use of convalescent plasma can have a therapeutic role, mainly because it is feasible and inexpensive. However, a small number of clinical results on the treatment with convalescent plasma is currently available. In particular, a meta-analysis was conducted in 2015 (J Infect Dis. 2015 Jan 1;211(1):80-90.doi: 10.1093/infdis/jiu396) with the main scope to evaluate the clinical efficacy of the administration of plasma or serum for the treatment of acute respiratory infections with viral etiology in order to be able to clinically manage MERS-CoV infection. Despite the fact that the studies analyzed were considered of low quality, with no control groups and with a moderate or high bias risk, the authors concluded that the administration of plasma or serum from immunized patients could be safely applied, as it was also characterized by a reduction of hospital stay and an overall decreased mortality. A possible explanation for the efficacy of this treatment is the reduction of the viraemia induced by the antibodies contained in the transfused convalescent plasma. The viral load usually peaks in the former week of infection and the patient develops a primary immune

response within days 10–14, followed by the clearance of the virus. For this reason, it may be more effective to administer convalescent plasma at the initial stage of the disease. More recently, the first evidence of a possible efficacy of the plasma therapy in patients with COVID-19 has emerged. In fact, a first study published in JAMA on March 2020 documented a positive response to the infusion of convalescent plasma in 5 patients admitted to the Intensive Care Unit in China (JAMA. 2020;323(16):1582-1589. doi:10.1001/jama.2020.4783). A further Chinese study performed in 10 patients confirmed the potential benefit of plasma therapy with an improvement of the prognosis of COVID-19 patients (Proc Natl Acad Sci U S A. 2020 Apr 28;117(17):9490-9496. doi: 10.1073/pnas.2004168117).

The availability of plasma donors allows the creation of a specific acquired immunity against the local viral strain of the infectious agent, which, in turn, could change over time as compared to the wild type strain. The possibility of collecting plasma by plasmapheresis procedure, thus making it immediately available for the patients care, currently represents an additional therapeutic option. The early use of plasma could play an important role in reducing the risk of ARDS and ICU transfer. The hypothesis of the present study is that the early use of convalescent plasma can reduce the need of mechanical ventilation, improve the survival and reduce the length of hospital stay of the COVID-19 patients. Furthermore, we aim to explore the time to get negative nasopharyngeal swab in patients treated with convalescent plasma.

To date no studies are present in the literature demonstrating its feasibility and effectiveness in the field of the worldwide SARS-CoV-2 pandemic.

OBJECTIVES

Primary objective

Primary objective of the study is to evaluate the efficacy of plasma obtained from convalescent COVID-19 donors and early administered to COVID-19 patients. The efficacy will be evaluated as the decrease in the percentage of patients who need mechanical ventilation (defined as first occurrence of a P/F ratio value <150 within 30 days from the randomization of the patient), or death within 30 days

Secondary objective

Secondary objectives include:

- mortality rates at 30 days
- time (days) to mechanical ventilation defined as reduction of PiO2/FiO2 <150 or death
- time (days) to mechanical ventilation or death
- time (days) to virological recovery (2 consecutive negative nasopharyngeal swabs)
- duration of hospitalization
- safety
- variation of SOFA score (Sequential Organ Failure Assessment)

STUDY DESIGN

Randomized controlled multicenter, national, open label, no-profit study

Recruitment of convalescent donors

Convalescent donors will be recruited by the participating Transfusion Services (TS), where the candidate donors will be identified. All donations through plasmapheresis will be performed at individual TSs following the "Operating protocol for the selection of convalescent patient-donors with a virologically documented diagnosis of COVID-19, for the biological qualification of the plasma from apheresis possibly produced as well as for subsequent correlated procedures for the reduction of pathogens and controlled storage ", established by the National Blood Center (Annex 1).

Duration of the study:12 months

STUDY POPULATION

The study will be conducted in hospital setting and will include patients selected on the basis of the criteria described below.

ELIGIBILITY CRITERIA

Main inclusion criteria for donors

- age 18-65 years
- confirmed diagnosis of previous COVID-19 disease
- Recovery from COVID-19 infection according to clinical features and laboratory molecular tests criteria specified by the guidelines released by the Italian Ministry of Health on 12 October 2020: negative nasopharyngeal swab after at least 10 days from symptoms onset (not considering anosmia and ageusia/dysgeusia) and at least 3 days without symptoms.
- in case of previously hospitalized donors, it is recommended to perform the apheresis after at least 28 days from the documented recovery.
- presence of adequate levels (>1:160) of neutralizing antibodies against SARS-COV-2
- informed consent signature

Main inclusion criteria for recipients

- Age≥18 years
- patients positive for SARS-CoV-2 diagnosed by RT-PCR performed on nasopharyngeal swabs or on deep breathing samples, pneumonia diagnosis since ≤10 days based on the following definitions:
    - Suggestive radiologic imaging (TC, RX, Ecography)

Donor exclusion criteria

- age <18 years or> 65 years
- other criteria according to the "Operational protocol for the selection of convalescent patient-donors with documented virological diagnosis of COVID-19, for the biological qualification of plasma from apheresis possibly produced as well as for the subsequent related procedures for the reduction of pathogens and controlled storage ", issued by the National Blood Center and attached to the present study protocol (Annex 1).

Inclusion Criteria for recipient patients (all the following ones):

- Age ≥ 18 years

- positive RT-PCR test for SARS-CoV-2 (nasal swabs or lower respiratory tract sample), with pneumonia diagnosis ($\leq$ 10 days) according to the following definitions:

- Suggestive radiological imaging (CT, RX, ultrasound);
- Respiratory failure not fully explained by heart failure or fluid overload;
- PaO2/FiO2 200-350 mmHg;

- Signed informed consent (the informed consent can also be orally collected in case the signature cannot be provided)

Exclusion Criteria for recipient patients (at least one of the following)

- need for non-invasive or invasive (CPAP/NIV) mechanical ventilation at randomization;
- PaO2/FiO2 <200;
- patients with hypersensitivity or allergic reaction to blood products or immunoglobulins;
- patients who clearly refuse to adhere to the clinical study;
- treatment with IL-6 receptor inhibitors, IL-1 inhibitors, JAK inhibitors, TNF inhibitors;
- participation in other clinical trials

Withdrawal Criteria

Withdrawal of consent

INTERVENTIONS

Plasma collection from convalescent donor

The plasma donation by plasmapheresis (volume from 600 to 700 mL, excluding the anticoagulant), must be carried out in compliance with current transfusion regulations. Furthermore, the indications reported in the Operational Protocol for the selection of convalescent patients-donors with documented virological diagnosis of COVID-19 have to be strictly followed for the biological qualification of plasma produced through apheresis. Also, the subsequent related procedures for the reduction of pathogens and controlled storage, drawn up by the National Blood Center and attached to this study protocol, have to be strictly followed as well.

According to the current transfusion legislation, the plasma donation should observe the following steps:

1) Acceptance of the convalescent donor according to the current transfusion regulations

The registration of the donor's personal data is carried out by the staff of the participating TSs through

IT management systems with procedure compliant with current blood transfusion regulations, prior to the acquisition of consent to the processing of personal data by the donor/convalescent. In addition to what already foreseen in the protocol, the following manners of collecting the informed consent are allowed:

a. Informed consent obtained in compliance with the National Blood Center authorization protocol N 0577 CNS 2020 for the plasma collection through apheresis in Coronavirus COVID-19 convalescent patients who recovered from the disease according to the clinical and laboratory criteria defined by the Ministry of Health on 12 October 2020, in order to transfuse the hyper-immune plasma in patients in ‘critical stage’,
b. Informed consent for plasma collection through apheresis from Coronavirus COVID-19 convalescent patients who recovered according to the clinical and laboratory criteria defined by the Ministry of Health on 12 October 2020, in order to transfuse the hyperimmune plasma in patients for a research scope.
c. Informed consent for plasma collection through apheresis from Coronavirus COVID-19 convalescent patients who recovered according to the clinical and laboratory criteria defined by the Ministry of Health on 12 October 2020, in order to transfuse the hyperimmune plasma in eligible patients, orally collected by phone from the donor in the presence of third parties (witnesses).

The IT management system generates an identification code univocally associated with the donation for each convalescent patient-donor registered. The donation is identified with a unique code, compliant with the transfusion regulations and reported in the identification label.

2) Selection of the convalescent donor

The donors' selection is carried out by the TS clinicians according to the indications of the Operational Protocol established by the National Blood Center and attached to this study protocol (Annex 1).

3) Collection procedure using a cell separator

The convalescent donor, duly informed about the apheresis procedure, agrees to sign the informed consent to donation. The collection of hyperimmune plasma will be performed through use of a cell separator supplied to the TS. To avoid any contact, the convalescent donor apheresis will be performed at different times as compared to the ones of the usual donors. Nursing assistance will be always guaranteed.

4) Biological qualification of hyperimmune plasma

See Operating Protocol drawn up by the National Blood Center and attached to this protocol of Study (Annex 1).

5) Procedure of pathogen reduction, freezing and storage of hyperimmune plasma

Each plasmapheresis will be aliquoted in sub-units of volume between 200 and 300 mL. Pathogen reduction, freezing and storage of hyperimmune plasma will be carried out, in compliance with the indications of the transfusion regulations and according to the validated operating procedures currently present at participating TSs.

In accordance with the national regulations, each plasmapheresis will be stored separately from other units collected for clinical use or industrial fractionation. If requested by the participating clinical site, the hyperimmune plasma units can be stored at + 4 ° C (± 2 ° C) and administered within 24 hours of collection.

6) Distribution of the blood component to the participating clinical sites

The assignment of fresh frozen plasma to the patient-recipient (according to study inclusion criteria and in accordance with the randomization procedure in use at all the clinical centers participating in the study) and the subsequent transfusion of the aforementioned blood component will take place in full compliance with current legislation regarding transfusion safety, traceability and haemovigilance.

Assignment to the patient (in accordance with the randomization procedure available for all participant clinical sites) and delivery to the clinical site will be tracked through the IT management

systems of the participating TSs. The exact dose of plasma to be infused (in the range of 200-300 mL for a maximum of 3 times over the period of 5 days) will be decided based on the characteristics of each patient. In order to monitor the viremia and the immune response, 2 peripheral blood samples will be collected without anticoagulant and 2 in EDTA immediately before and 24 hours after the hyperimmune plasma infusion.

EVALUATION OF THE POTENTIAL BENEFIT/RISK RATIO FOR THE POPULATION

The potential risks for the study population are the followings:

- for donors: possible undesirable reactions, immediate or late, related to the donation of plasma by apheresis.
- for recipients: possible mild, moderate or severe side effects correlated to the plasma transfusion.

Assignment to the experimental group:

Patients who meet the inclusion criteria will be enrolled by the clinician in charge and, through the GIMEMA platform, will be randomized in a 1: 1 ratio to one of the following groups:

a) administration of hyperimmune plasma in addition to standard therapy. The exact dose of plasma to be infused will be decided based on the clinical condition of each patient. A range of 200-300 ml for a maximum of 3 times within 5 days is recommended.

b) standard therapy (see below)

Withdrawal of subjects and modifications of the intervention

The withdrawal of the subjects will be possible in case of withdrawal of the informed consent.

The intervention is suspended in case of correlated adverse events

Standard therapy

Regardless of the assigned arm, all patients will continue to receive standard therapy, including that one approved for the SARS-CoV-2 infection. Standard therapy will be carried out in accordance with the AIFA indications with regard to the choice of drugs, dosages and durations of treatment.

The concomitant use of IL-6 receptor inhibitors, IL-1 inhibitors, JAK inhibitors, TNF inhibitors is not allowed starting from the randomization.

Rescue Therapy

Patients will be monitored according to normal clinical practice. In case of worsening of the clinical conditions, the Investigator can decide to administer other drugs as rescue therapy.

Early termination of the study

The Sponsor may interrupt the study at any time and promptly notify the investigators and ethics committees. Patients will be evaluated as soon as possible and continuously followed up according to the normal clinical practice.

Definition of study conclusion

The patient will be followed up for 30 days after plasma administration (30 days follow-up) in order to evaluate the possible occurrence of adverse events. The end of the study will be coincident with the end of follow-up of the last patient enrolled.

ENDPOINT OF THE STUDY

Primary endpoint

In agreement with the primary objective, the efficacy will be measured by calculating the reduction in the percentage of patients who meet the need for mechanical ventilation (defined as the first occurrence of a P/F<150 value within 30 days from the patient randomization) or death within 30 days.

Secondary endpoints

- Death at 30 days
- Time (days) to invasive mechanical ventilation defined as reduction of P/F<150 or death
- Time (days) to invasive mechanical ventilation or death
- Time (days) to virological recover (2 consecutive negative nasal-pharingeal swabs)
- Duration of hospital stay

- SOFA score variation (Sequential Organ Failure Assessment)
- Safety related to eventual adverse events onset

Ancillary endpoint

- Evaluation of the lymphocyte asset (CD4/CD8 ratio and CD8 activation at day 0, 3, 7, 14 from the treatment).

STUDY PLAN

Timeline of the study

Enrollment period: May 2020 - March 2021 (expected conclusion of the study 31 May 2021)

Assessments

The P/F evaluation will be measured daily

For all the other measurements, please refer to the attached time schedule (Appendix 1).

Sample size

In the light of the published results, the occurrence of the defined clinical event in the absence of treatment (need of mechanical ventilation, defined as first occurrence of a P/F<150 value within 30 days from the patient randomization or death) was estimated in 30% of patients (P0 = 0.30). The study sample size was calculated to highlight a 40% reduction in the primary endpoint (delta = 0.12; P1 = 0.18), with a statistical power of 80% and an error alpha of 5%, by using a two tailed statistical test for comparison of two proportions, and a 1: 1 randomization ratio. By adopting a sequential design with 2 interim analyses, it is planned to conduct the first interim analysis after the enrollment of 120 patients, a second interim analysis after the enrollment of 238 patients and the final analysis with 474 patients. Details on sample size calculation are available in the Study plan section (see below).

THE CALCULATIONS WERE CARRIED OUT USING THE RPACK SOFTWARE (CONFIRMATORY ADAPTIVE CLINICAL TRIAL DESIGN AND ANALYSIS, R PACKAGE).

To take into account that the prognosis of the disease changes significantly over time, at the time planned for the conduction of the first interim analysis (conclusion of the follow-up at 30 days of the first 120 patients enrolled), before conducting the interim analysis itself, the sample size assumptions of the study will be evaluated [according to EMA "Reflection paper on methodological issues in confirmatory clinical trials planned with an adaptive design" (CHMP/EWP/2459/02)]. The evaluation will be based on the proportion of events observed only in the standard therapy group and will follow the following decision algorithm:

Observed P0 in the control group (at the moment of the first interim analysis)	Conduction of first planned interim analysis (n=120 patients)	Sample size re-calculation
≥0.26	YES	NO
0.19-0.259	YES	YES
<0.19	NO	YES

– If the proportion of observed events in the control group (P0) is <19.0%, the study will be re-sized to redefine the two interim analyses and the final analysis. The value P0=19.0% corresponds to the lower limit of a hypothetical 95% confidence interval of the proportion=30% calculated in the control group (n.~60) at the first interim analysis. In addition, for values of P0<19%, with a number of 60 patients per arm, the minimun detectable effect (MDE) for a p-value=0.0208 (alpha allocated for the first interim analysis) would be greater than 75% (corresponding to P1<5%), above the parameters set in the planning phase of the study, so it is not considered appropriate to spend the alpha and it becomes necessary to redefine the size of the study.
– If P0 in the control group is between 19.0% and 25.9% (ensuring an MDE between 67% and 75%) the first interim analysis will be carry out as pre-specified in the protocol and the sample size of the study to define the second interim analysis and the final analysis will be calculated
– If P0 in the control group is greater than or equal to 26.0%, the first interim analysis will be carry out as per protocol, subsequent analyses will remain unchanged, and the study will not be re-sized.

Change in the number of interim analyses.

- As a consequences of the high enrolment rate observed in November 2020, trial patient enrolment reached the planned sample size before the number of patients needed for the second interim analysis completed the follow-up period. Therefore, it was decided - as the recommended by the DSMB - not to conduct the second interim analysis because the results of this analysis should not have impacted the enrollment (already completed), nor the treatment of patients (already all treated), nor should it have significantly anticipated the final outcome (given the brevity of the remaining follow-up to complete the observation of all patients in the study).

DATA MANAGEMENT

Data collection

Data collection will be carried out continuously (daily) for the entire duration of the treatment. The data will be uploaded on the database developed on REDCap (a tool for collecting data from clinical study). The data will be recorded via electronic Case Report Forms (eCRF) and uploaded in the REDCap system. The collection of the data related to the primary endpoint will be monitored during the course of the study in order to reduce the number of missing data.

Data management

The GIMEMA Foundation will be in charge of the data management of the study (see Appendix 2: Management and implementation of the clinical trial in accordance with the study protocol). All data will be pseudoanonymized. The processing and data storage will be performed in compliance with the law on personal data protection (Legislative Decree 30/6/2003 n. 196, Guidelines for the processing of personal data in the context of clinical trials - 24 July 2008 - G.U. n. 190 of 14 August 2008 and the EU Regulation 2016/679, known as GDPR (General Data Protection Regulation).

The investigators who take care of the patient are responsible for the patient data and for obtaining the informed consent. Consent may be oral if it is not possible to obtain a written consent. If the subject is unable to provide the informed consent and an authorized representative is not available, the treatment can be administered without consent without any delays if the Investigator evaluates that the treatment can have a potential life-saving effect. Consent to remain in the study must be acquired as soon as the patient is able to agree. The same procedure applies to patient information, consent to processing personal data in accordance with the European Regulation n. 679/2016 on the protection of personal data, code of protection of personal data (legislative decree 196/03) and subsequent amendments and additions, as well as to the provisions, guidelines and general authorizations of the national supervisor for the protection of personal data.

Data retention

In agreement with the mandatory retention time defined by the law, the pseudoanonymized data will be stored in the REDCap platform for a period not exceeding the one established for the purposes of the study and for which data were collected (and in any case no later than 10 years from conclusion of the study). Every personal data will be deleted after the applicable retention period expires. However, the participating subjects have the right to request, at any time, the deletion of data, in compliance with the GDPR and the applicable data protection law. In compliance with the privacy legislation, the data will be sent to the Center promoting and coordinating the study to be processed and analysed.

Administrative aspects

The study is not for profit. The stipulation of a suitable insurance policy is required.

STATISTICAL PLAN

The primary analysis will be conducted in the Intention To Treat (ITT) population. In the primary analysis, the percentages of subjects who will need mechanical ventilation (defined as the first occurrence of a P/F<150 value) or death within 30 days from randomization) in the two

experimental groups will be compared with the 2-tailed chi-square test. The proportions observed in the two groups will be presented with relative 95% confidence interval (95% CI). Categorical variables will be described as frequencies and percentages, quantitative ones with mean and standard deviation if normally distributed or median and interquartile range if not normally distributed. Comparisons of the two groups for time to event data will be carried out by logrank test and Cox model The time course of the parameters indicating the viral load and the immune response they will be studied with models for repeated measures over time. The proportion of grade ≥3 adverse events will be described, together with 95% CI. An ad hoc SAP will be prepared which will include all the procedures for the treatment of any missing data and any sensitivity analyzes.

Calculation of the sample size (Appendix 3)

Considering the daily provided and published data, mechanical ventilation in the absence of treatment is needed in 30% of patients (P0 = 0.30). The study size was calculated to highlight a 40% reduction in the primary endpoint (delta = 0.12; P1 = 0.18). A statistical power of 80% and an alpha error of 5% were considered, with two-tailed statistical test for comparison between two proportions and a 1: 1 randomization ratio. Based on these assumptions, adopting a sequential design with 2 interim analyzes, the Pocock method for control of the family-wise type I error, and using information rates of 0.25 and 0.50 for the first and second interim analyses respectively, it is planned to conduct the first interim analysis with 120 patients enrolled, the second interim analysis with 238 enrolled patients and a possible final analysis with 474 patients. With these specifications it will be possible to highlight a minimum Detectable Effect (MDE) of about 60%). Based on the observed enrollment rate, it will be possible decide to unbalance the randomization ratio in favor of the plasma group (2: 1). If so, sample size will be recalculate accordingly.

Calculations were conducted using RPACK (Confirmatory Adaptive Clinical Trial Design and Analysis, R package).

Randomization

Being an unblinded, parallel, multicentre study, the assignment of a patient to one of the two arms will take place through a centralized block randomization procedure with a stratification factor for

clinical center. The randomization list will be generated by the coordinating center via validated electronic procedure by STATA ® software After verifying the presence of the inclusion / exclusion criteria the investigator will assign a sequential randomization code. Patient randomization will be edited by GIMEMA on the basis of the randomization list sent by the coordinating center.

Pairing procedure

In the event of difficulty in recruiting, even following the possible introduction of the rate of randomization of 2: 1, or the non-feasibility of the randomized study, the advisability of follow a retrospective cohort approach (albeit with less solid foundations) by identifying an appropriate group of controls, following an individual pairing to the patients enrolled in the experimental arm. Each patient belonging to the experimental arm will be paired (1: 1) with another patient of a cohort retrospective of inpatients in the same center and in a near period of time. The matching will be performed considering the center, age, sex, hospitalization period, severity, as matching variables, and possible concomitant therapies among those provided as standard of care. This analysis will be pre-specified with all the statistical details in an amendment to the protocol before the conclusion of enrollment and access to data by defining the pairing criteria and endpoints (primary and secondary) that will be possible compare. As a further supportive analysis (limited to the mortality endpoint), a

comparison with survival curves of the hospitalized population of the ITA-COVID network.

Following a counterfactual approach, each patient treated in the experimental arm will be assigned one expected mortality (at fixed time point) equal to that (for the same sex and age characteristics) of the ITACOVID cohort thus defining the expected mortality of a hypothetical control group with the same characteristics by age and sex of randomized patients. A comparison will then be made between the expected mortality and observed.

SAFETY MANAGEMENT

Adverse event reporting

The investigator will enter in the REDCap platform any adverse event or laboratory results no later than 24 hours after the occurrence of the event. If necessary, the Promoting Center may request the investigator to follow up on the event.

Reporting of adverse reactions to plasma

In accordance with the EMA GVPs, the recording of each adverse reaction is foreseen, (https://www.ema.europa.eu/en/humanregulatory/postauthorisation/pharmacovigilance/goodpharmacovigilance-practices), for both plasma donors and recipients. Such reactions will be recorded using the National Information System of Transfusion Services (SISTRA) related to haemovigilance.

Reporting of adverse drug reactions

In case a suspected adverse reaction to drugs used for standard therapy is observed, and in accordance with Regulation (EU) 536/2014, the report must be registered in the Clinical Trial module of Eudravigilance.

DATA SAFETY AND MONITORING BOARD

It will be created a Data Safety and Monitoring Board (DSMB) which will periodically review the data related to the patient safety and the conduction and evolution of the study. In addition, the DSMB will provide recommendations regarding the prosecution, modification or premature conclusion of the trial.

ETHICAL CONSIDERATIONS

The study will be conducted in accordance with the ethical principles established in the latest revised version of the Helsinki Declaration. Participation in the study will be subject to obtaining free and informed consent. The rights established in the law regarding the protection of personal data of the subjects included in the study will be safeguarded (General EU regulation on the protection of personal data 679/2016; Legislative Decree 30/6/2003 n. 196 and subsequent amendments; Requirements related to the processing of personal data carried out for the purposes of scientific research by the Authority for the protection of personal data - aut. gen. n. 9/2016).

Acquisition of informed consent

Informed consent will be acquired through an approved informed consent form from all patients included in the study, including unable ones or in emergency cases. Consent can be oral if not it is possible to express a written consent. If the subject is unable to provide the informed consent and an authorized representative is not available the treatment can be administered without consent without any delays when the Investigator evaluates that the treatments itself can have a potential life-saving effect. Consent to remain in study must be acquired as soon as the patient is able to agree.

Confidentiality

All information on personal data of eligible subjects collected before, during and after the trial conclusion will be processed confidentially, in accordance with current legislation.

Conflict of interest

No conflict of interest

APPENDIX 1: TIME SCHEDULE OF THE STUDY

	Arruolamento	Infusione Plasma^			Follow-up dal giorno della randomizzazione				
	Giorno 0	Plasma 1	Plasma 2	Plasma 3	Giorno 1	Giorno 3	Giorno 7	Giorno 14	Giorno 30
Checklist<br>- Criteri di inclusione<br>- Criteri di esclusione<br>- Consenso informato	X								
**Demografica**	X								
**Random**	X								
**Dati relativi al ricovero**	X								
**Patologie concomitanti/precedenti**	X								
**Terapie concomitanti per patologie diverse da COVID-19**	X				X	X	X	X	X
**Valutazione**<br>- Esami ematochimici<br>- Segni vitali<br>- Scheda parametri di funzionalità respiratoria<br>- Emogasanalisi	X				X	X	X	X	
**Assetto linfocitario**	X					X	X	X	
**Ossigenoterapia**					X	X	X	X	X
**Terapia Standard**					X	X	X	X	X
**Infusione Plasma**		X	(x)	(x)					
**Tampone**						X	X	X	X
Outcome<br>– PaO2/FiO2<150<br>– Decesso<br>– Ventilazione meccanica invasiva<br>– Dimissione<br>– Trasferimento in altro reparto/struttura									
**Terapia rescue**									
**Eventi avversi**									
**Off (uscita dallo studio)**									

^ un massimo di 3 volte nell'arco di 5 giorni

APPENDIX 2: MANAGEMENT AND IMPLEMENTATION OF THE CLINICAL TRIAL IN ACCORDANCE WITH THE STUDY PROTOCOL

GIMEMA Foundation will be in charge, through a delegation letter, to collaborate and support the Sponsor with all the operative services useful to run the multicentric clinical study as following described.

Documentation – In strict collaboration with the Sponsor, GIMEMA will support to preparing the needed trial documents, such as: 1) SOPs (in compliance with the ICH and GCP qualitative standards), 2) patient informed sheet and informed consent for both donors and recipients, 3) monitoring manual, 4) training material, 5) trial master file. In collaboration with the Sponsor, GIMEMA will prepare the documents useful for the local Ethic Committees and it will be in connection with them. In addition, it will prepare all the needed documentation to open the clinical sites.

Data management – GIMEMA will take on the management of the collected data in agreement with the protocol objectives through a database developed in REDCap. The data needed for the study will be registered through electronic Case Report Forms (eCRF). In particular, ad hoc eCRF will be prepared for both donors and recipients. The system has been validated by the Data Center of the GIMEMA Foundation according to the CAMP5 and GCP IC E6 (R2) criteria and it has been installed on an IT infrastructure according to ISO27001:2013, ISO 27017:2015 and ISO 27018:2014 standards. The system allows to develop CRF accessible to the investigators by web. It also guarantees the data integrity by registering each user activity and also ensuring to not hiding the data and an adequate audit trail.

The GIMEMA Data Center is ISO9001:2015 certified and it is also a certified ECRIN (European Clinical Research Infrastructure Network) Data Center. The ECRIN certification program identifies not commercial Clinical Trial Units in Europe that demonstrate to provide a safe, trustworthy, and efficient clinical research management of data also in compliance with the current regulation.

All the data will be pseudonymized and each Investigators will be the only ones knowing the relation between the code of the enrolled subject and his/her identity. The processing and storage of the data will be in compliance with the laws related to the personal data protection (Legislative Decree 30/06/2003 n.196; Guidelines for processing the personal data within the drug clinical

experimentation – 24 July 2008 – G.U. n.190, 14 August 2008) and the UE Regulation 2016/679, also known as GDPR (General Data Protection Regulation).

When a clinical site will be opened the authorized users will receive all the needed credentials from the Help Desk of the Data Center of the GIMEMA Foundation in order to access the eCRF. The management of the access privileges of the users is up to the Help Desk.

As foreseen in the study protocol, the assignment of the patients to one of the two trial arms will be done through a centralized procedure of randomization. The patient randomization will be performed by GIMEMA based on the randomization list prepared by the coordinating center.

The authorized users will receive an instruction manual useful to accessing and compiling the forms. Manuals and tutorials will be available online together with all the technical assistance key contacts.

GIMEMA will take care of the database hosting through REDCap.

Registration procedure for donor patients – A donor patient can be registered after the Investigators have checked that all the eligibility criteria are accomplished. This can be done through a checklist form available in the eCRF on REDCap.

Registration procedure for the study patient – A patient eligible for the plasma donation can be registered after the Investigators have checked the eligibility criteria are accomplished. This can be done through a checklist form available in the eCRF on REDCap. A not registered patient before the beginning of the treatment cannot be enrolled in the study.

Data flow – The Investigator has to verify that the CRF are compiled as soon as possible, and also completely and properly inserted in the REDCap system. The GIMEMA Data Center will deeply control with regard to the coherence of the inserted data and it can issue electronic Query (integrated in the REDCap system) when the data are incoherent or missing. The Investigator (or the authorized personnel of the clinical site) can electronically reply always through the platform. The GIMEMA Data Manager will further check the answers and eventual data modification. This flow is tracked by an audit trail contained into the system.

Monitoring of the clinical sites – When foreseen in the protocol, GIMEMA will perform the remote monitoring of the clinical sites thanks to its expert CRAs carefully trained.

APPENDIX 3: Calculation of the sample size

Parameters used in the calculation of the sample size:

- H0: P0 = 0.3
- H1: P1 = 0.18 (delta = 0.12)
- alpha = 5%
- Power = 80%
- two-tailed test
- Sequential design with 2 interim analyses
- Pocock's method for checking the family-wise type I error
- Information rates of 0.25 and 0.50 for the first and second interim analysis respectively

Summary Table

```
Sample size calculation for a binary endpoint

Sequential analysis with a maximum of 3 looks (group sequential design).
The sample size was calculated for a two-sample test for rates (two-sided),
treatment rate pi (1) = 0.18, control rate pi (2) = 0.3, allocation ratio = 1, and power 80%.

Stage                                      1      2      3
Information rate                         25%    50%   100%
Efficacy boundary (z-value scale)      2.312  2.312  2.312
Number of subjects                       119    237    474
Cumulative alpha spent                0.0208 0.0358 0.0500
Cumulative power                      0.2182 0.4755 0.8000
Two-sided local significance level    0.0208 0.0208 0.0208
Lower efficacy boundary (t)           -0.174 -0.128 -0.092
Upper efficacy boundary (t)           -0.174 -0.128 -0.092
Exit probability for efficacy (under H0) 0.0208 0.0151
Exit probability for efficacy (under H1) 0.2182 0.2573

Legend:
  (t): approximate treatment effect scale
```

Minimum Detectable Effect (MDE)

Analysis	Interim 1	Interim 2	Final
**MDE**	**-0,174**	**-0,128**	**-0,092**
**Difference %**<br>**(vs 30%, control group)**	**58%**	**43%**	**30%**

R command

```
design <- getDesignGroupSequential(typeOfDesign = "P", informationRates = c(0.25, 0.5,1), alpha = 0.05,
sided = 2)
print(design)
getDesignCharacteristics(design)
designPlan <- getSampleSizeRates(design, pi1 = 0.18, pi2 = 0.3)
summary(designPlan)
print(designPlan)
```

Plot 1: Rate Difference

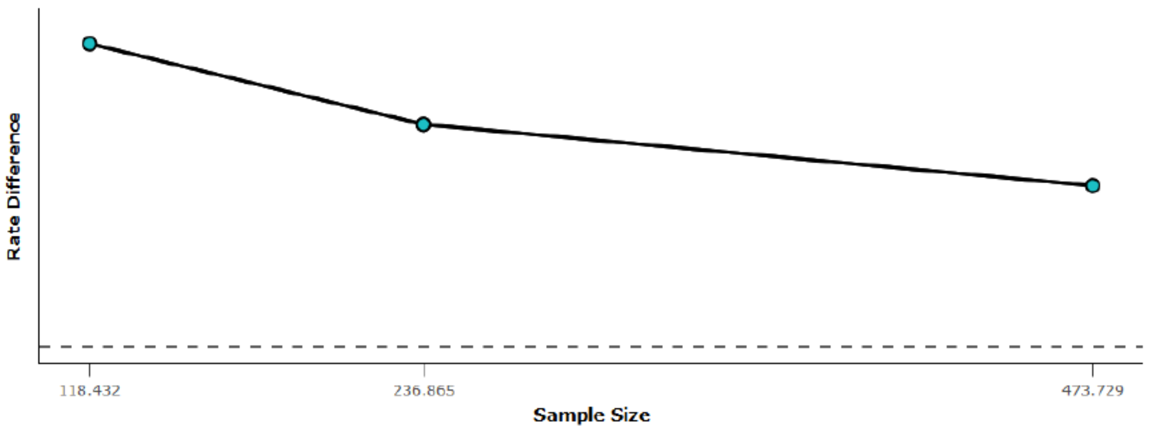

Plot 2: Cumulative alpha spending

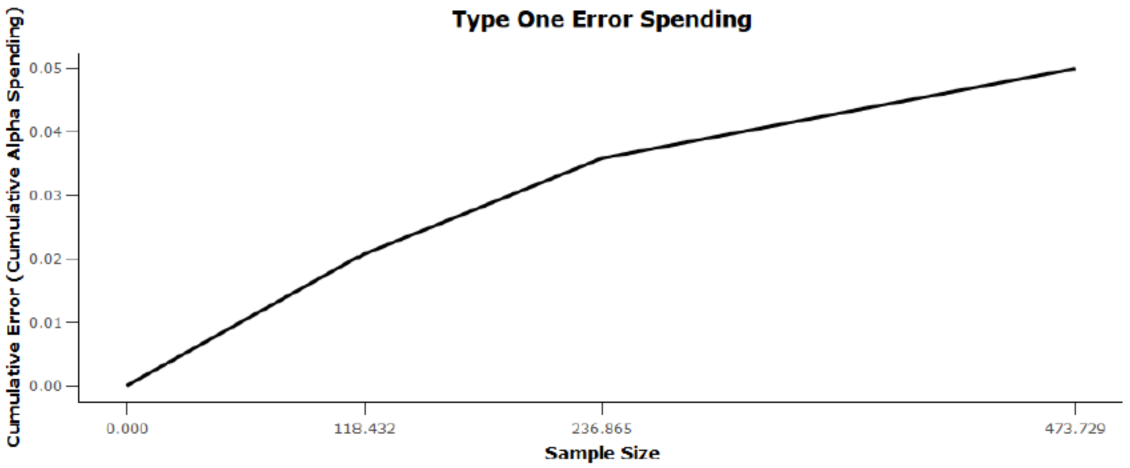

TSUNAMI Study - Annex 1:
Operating protocol for the selection of convalescent patient-donors with a virologically documented diagnosis of COVID-19, for the biological qualification of the plasma from apheresis possibly produced as well as for subsequent correlated procedures for the reduction of pathogens and controlled storage.

Based on knowledge to date, convalescent plasma therapy is to be considered 'empirical' and not supported by robust scientific evidence and solid haemovigilance data on its safety. However, the COVID-19 pandemic is a typical situation where plasma from convalescents can be a resource to support treatment of a disease in clinical trials and observational studies, as a readily available, low-risk experimental therapy[1,2].

Therefore, in view of the possible current clinical use and possible future developments, the following conditions are to be met for the selection of convalescent donor-patients with a virologically documented diagnosis of COVID-19. All convalescent donor-patients must however be subject to a careful clinical and anamnestic assessment, even in the event of them being accepted when the current selection criteria for blood and blood component donors are not met.

1. Patient–donors, with virologically documented diagnosis of COVID-19, completely recovered according to the clinical and laboratory criteria defined by the Superior Health Council on 12 October 2020[3] (one negative SARS-CoV-2 molecular swab test). Plasma collection (donation) can occur 28 days after the documented recovery;
2. Male patient–donors or a nulliparous female donors with a negative history of blood component transfusion;
3. Careful clinical evaluation of patient–donors with particular reference to the criteria provided for under the current regulations to protect the health of apheresis donors;
4. Negative results of the biological qualification tests in compliance with the provisions of the regulations in force;
5. Presence of adequate levels of anti-SARS-CoV-2 neutralising antibodies (VNT $\geq$ 1:160).

In addition, each unit of plasma apheresis collected from convalescent patient–donors must be:

1. processed with a pathogen reduction method of recognised efficacy;
2. clearly labelled as 'Plasma unit collected from a convalescent patient–donor with a virologically documented diagnosis of COVID-19';
3. kept separately from other units for clinical use or industrial fractionation;

[1] World Health Organization (WHO) Blood Regulators Network, September 2017. Disponibile all'indirizzo web: https://www.who.int/bloodproducts/brn/2017_BRN_PositionPaper_ConvalescentPlasma.pdf?ua=1.
[2] European Commission. Guidance document on the collection and transfusion of convalescent COVID-19 plasma. Disponibile all'indirizzo web: https://ec.europa.eu/health/blood_tissues_organs/covid-19_en.
[3] Ministero della Salute. Direzione Generale della Prevenzione Sanitaria e Direzione Generale della Programmazione Sanitaria. Circolare "COVID-19: indicazioni per la durata ed il termine dell'isolamento e della quarantena" Prot. n. 0032850-12/10/2020-DGPRE-DGPRE-P del 12/10/2020.

Centro Nazionale Sangue
Via Giano della Bella, 27 - 00162 Roma
Tel: +39 06 4990 4953 / 4963
Email: segreteriagenerale.cns@iss.it

kiwa certified
SISTEMA DI GESTIONE CERTIFICATO
UNI EN ISO 9001:2015

4. aliquoted in sub-units with a volume of between 200-300 mL; given the impossibility of refreezing sub-units once thawed if not used, they can be stored at 4 ± 2°C for a period of no more than 5 days, after which unused units must be discarded;
5. issued for clinical use in compliance with the provisions of the regulations in force (Ministerial Decree of 2 November 2015), regarding the adoption of measures to ensure transfusion safety.

Finally, health operators are strongly advised to:

1. collect, at the time of donation, a plasma sample which, aliquoted and appropriately stored, can be used for possible subsequent studies related to COVID-19;
2. manage the exchange of information regarding patient-donors in accordance with the current legislation on the protection of personal data, and to appropriately adopt the informed consent of patient-donors and of the possible recipient provided for in the study protocol;
3. keep the National Blood Centre and the competent Regional Coordination Centre for transfusion activities informed about the possible start of collection procedures, as well as about the number and volume of units that may be collected and made available (differentiated by blood group phenotype);
4. strengthen the haemovigilance activities already provided in accordance with current legislation for adverse reactions in donors, adverse reactions in recipients and adverse events, in order to comply with the national and European information flow;
5. promptly notify adverse reactions/events to the competent regional and national authorities, after ensuring that they are clearly attributable to the donation and/or transfusion of COVID-19 convalescent plasma.
