# Supplementary material for: Effect of High-Titer Convalescent Plasma on Progression to Severe Respiratory Failure or Death in Hospitalized Patients With COVID-19 Pneumonia: A Randomized Clinical Trial
Source: JAMA Netw Open. 2021 Nov 29;4(11):e2136246. doi: 10.1001/jamanetworkopen.2021.36246 (PMC8630572; doi:10.1001/jamanetworkopen.2021.36246)
Supplement: Supplement 2. — eAppendix. Statistical Analysis eTable 1. Characteristics of the Patients at the Baseline in the Randomized Population eTable 2. Convalescent Plasma Treatment During Trial, mITT Population eTable 3. Oxygen Supplementation and Treatment During Trial (Day 1 to Day 30 Post Randomization) in the mITT Population eTable 4. Characteristics of the Patients at the Baseline in the ITT Population eTable 5. Primary End Point in the ITT Population eTable 6. Primary End Point in the mITT Population According to Lag From Onset of Symptoms to Convalescent Plasma (CP) Infusion eTable 7. Secondary End Points in the mITT Population eFigure 1. Primary End Point Outcome According to Trial Group and Clinical Sites in the mITT Population eFigure 2. Survival Curves at 30 Days of Secondary Outcomes in the mITT Population eReferences. [file jamanetwopen-e2136246-s002.pdf]

Supplemental Online Content

Menichetti F, Popoli P, Puopolo M, et al; TSUNAMI Study group. Effect of high-titer convalescent plasma on progression to severe respiratory failure or death in hospitalized patients with COVID-19 pneumonia: a randomized clinical trial. *JAMA Netw Open.* 2021;4(11):e2136246. doi:10.1001/jamanetworkopen.2021.36246

eAppendix. Statistical Analysis

eTable 1. Characteristics of the Patients at the Baseline in the Randomized Population

eTable 2. Convalescent Plasma Treatment During Trial, mITT Population

eTable 3. Oxygen Supplementation and Treatment During Trial (Day 1 to Day 30 Post Randomization) in the mITT Population

eTable 4. Characteristics of the Patients at the Baseline in the ITT Population

eTable 5. Primary End Point in the ITT Population

eTable 6. Primary End Point in the mITT Population According to Lag From Onset of Symptoms to Convalescent Plasma (CP) Infusion

eTable 7. Secondary End Points in the mITT Population

eFigure 1. Primary End Point Outcome According to Trial Group and Clinical Sites in the mITT Population

eFigure 2. Survival Curves at 30 Days of Secondary Outcomes in the mITT Population

eReferences.

This supplemental material has been provided by the authors to give readers additional information about their work.

© 2021 Menichetti F et al. *JAMA Network Open.*

eAppendix. Statistical Analysis

Statistical analyses were conducted in accordance with the pre-defined statistical analysis plan. The following study populations were defined: the randomized (RND) population included all randomized patients; the intention to treat (ITT) population included patients as randomized excluding those who withdrawn informed consent; the modified intention to treat population (mITT) included patients who received allocated treatment (CP+ST and ST); the per protocol population (PP) included patients without major deviations (mechanical ventilation with PaO2/FiO2>150, rescue therapy, low-titer CP, interval between first and last CP infusion longer than 5 days).

Efficacy analyses were based on the modified intention to treat (mITT) population, and for primary endpoint also on the per protocol (PP) populations whereas safety analyses were carried out on the randomized (RND) population. Shapiro Wilks test was used to assess normal distribution of continuous data. Median, interquartile range (IQR) and Mann-Whitney test were used for not normally distributed continuous data; frequencies, percentages and chi-square test for categorical variables; Kaplan-Meier survival curves and log-rank test for time to event data [time to death/mortality, time to mechanical ventilation or death, time to virological clearance (defined by two consecutives negative nasopharyngeal swabs)] were calculated. CP+ST effect as compared to ST was reported as crude Odds Ratios (ORs) and 95% confidence intervals (CIs) estimated by univariate logistic regression models. Pre-specified subgroups (defined by baseline characteristics: age, gender, PaO_2/FiO_2, time from onset of symtpoms, use of antivirals, use of heparin, use of corticosteroids, anti-S-Ig antibody, NAbs titer, and comorbidities) analyses on primary endpoint were planned. In each subgroup, CP+ST effect as compared to ST was reported as crude Odds Ratios (ORs) and 95% confidence intervals (CIs). Differences among subgroups were assessed through test of interaction terms in properly defined multivariate logistic regression models[1]: for each baseline characteristic, the model included a dummy variable for the experimental group (CP+ST vs ST), a dummy variable for the baseline characteristic (or dummy variables for baseline characteristic with more than two

© 2021 Menichetti F et al. *JAMA Network Open.*

categories) and term(s) of their interaction(s), including clinical site (hospital level) as clustering factor.

Sensitivity analyses by Non Responding Imputation (NRI) and Tipping Point Analysis (TPA) were planned to handle missing data in primary endpoint. Post-hoc analyses on primary endpoint were performed according to time from onset to randomization (≤5 and 6+ days), NAbs titer of transfused CP (<320; 320+), lag (in hours) from randomization to first CP infusion (<=10 and 11-40 hours), clinical sites, and levels anti-S IgG test at baseline. All p-values were two-sided and the overall type I error of the trial was set at 0.05 (nominal alpha for the final analysis of the primary endpoint was 0.0292). The analyses were performed using STATA version 16.1.

Sample size and interim analysis

A three stage (two interim analyses and final analysis) group sequential design was planned. Sample size calculation was obtained by using R PACK (R Package for adaptive Clinical Trials), for a two-sample test for rates, Pocock method for control of family-wise type I error, and information rates of 0.25 and 0.50 for first and second interim analyses. By assuming that 30% of COVID-19 patients on ST would meet the composite primary outcome within 30 days from randomization, to shows a clinically significant reduction of 40% in the CP+ST arm with respect to ST group in the primary composite endpoint, with a power of 80% and significance level of 5%, a sample size of 237 participants in each arm (total 474 participants for the study) was estimated. Two interim analyses were planned at the conclusion of the follow-up period on 119 and 237 patients. The assumption that 30% of the participants in the control arm would meet the composite primary outcome was based on the best available evidence for Italy at the time the trial was designed[2]. Adequacy of this assumption for sample size calculation was assessed according to European Medicines Agency (EMA)[3], before conducting the first interim analysis.

© 2021 Menichetti F et al. *JAMA Network Open.*

eTable 1. Characteristics of the Patients at the Baseline in the Randomized Population

	Total<br>No (%)	Convalescent plasma<br>No (%)	Standard therapy<br>No (%)
	**n=487**	**n=241**	**n=246**
**Age**, median [IQR]-yrs	64.0 [54.0-74.0]	65.0 [55.0-74.0]	63.5 [54.0-74.0]
**Gender**			
Female	175 (35.9%)	87 (36.1%)	88 (35.8%)
Male	312 (64.1%)	154 (63.9%)	158 (64.2%)
**Race/Ethnicity**			
White	450 (92.4%)	223 (92.5%)	227 (92.3%)
Non-white[a]	11 (2.3%)	6 (2.5%)	5 (2.0%)
Asian	8 (1.6%)	5 (2.1%)	3 (1.2%)
Others	17 (3.5%)	7 (2.9%)	10 (4.1%)
Missing	1 (0.2%)	0 (0.0%)	1 (0.4%)
**BMI**, median [IQR]	26.0 [24.1-28.6]	26.0 [24.2-29.1]	26.0 [23.9-28.1]
**Time from onset of symptoms**, median [IQR] - days	7.0 [5.0-9.0]	7.0 [5.0-9.0]	7.0 [4.0-8.5]
**Comorbidities**			
None	102 (20.9%)	52 (21.1%)	50 (20.7%)
Hypertension	184 (37.8%)	87 (36.1%)	97 (39.4%)
Type 2 diabetes	94 (19.3%)	49 (20.3%)	45 (18.3%)
COPD	28 (5.8%)	14 (5.8%)	14 (5.7%)
Chronic renal failure	23 (4.7%)	7 (2.9%)	16 (6.5%)
Solid cancer	17 (3.5%)	10 (4.2%)	7 (2.9%)
Congestive heart failure	11 (2.3%)	5 (2.1%)	6 (2.4%)
**Smoking**			
None	322 (66.1%)	162 (67.2%)	160 (65.0%)
Previous tobacco use	108 (22.2%)	48 (19.9%)	60 (24.4%)
Current tobacco use	15 (3.1%)	9 (3.7%)	6 (2.4%)
Missing	42 (8.6%)	22 (9.1%)	20 (8.1%)
**Disease severity**, median [IQR]			
PaO2/FiO2	273.0 [238.0-308.0]	277.0 [237.0-305.0]	266.3 [240.0-309.0]
SOFA score[1]	2 [2-3]	2 [2-2]	2 [2-3]
**Previous treatments**			
Remdesivir	13 (2.7%)	5 (2.1%)	8 (3.3%)
Corticosteroids	100 (20.5%)	48 (19.9%)	52 (21.1%)
LMWH	94 (19.3%)	48 (19.9%)	46 (18.7%)

[1]missing data for 112 patients (52 CP+ST; 60 ST)

[a] Race: non-white included: Black or African American.

CP: convalescent plasma; ST: standard therapy; No: number of patients; IQR: interquartile range; COPD: chronic obstructive pulmonary disease; BMI: body-mass index; SOFA: Sequential Organ Failure Assessment; LMWH: low-molecular-weight heparin

© 2021 Menichetti F et al. *JAMA Network Open.*

eTable 2. Convalescent Plasma Treatment During Trial, mITT Population

	**Convalescent Plasma No (%)**
	**n=232**
**Number of infusions**	
1	35 (15.1%)
2	175 (75.4%)
3	22 (9.5%)
**NAbs titer***, median [IQR]	226.3 [160.0-320.0]
**NAbs titer[1] category**	
<320	149 (64.2%)
320+	83 (35.8%)

[1]geometric mean of infusion titers

CP: convalescent plasma; No: number of patient

© 2021 Menichetti F et al. *JAMA Network Open.*

eTable 3. Oxygen Supplementation and Treatment During Trial (Day 1 to Day 30 Post Randomization) in the mITT Population

	Total<br>No (%)	Convalescent plasma<br>No (%)	Standard Therapy<br>No (%)
	**n=473**	**n=232**	**n=241**
**Use of oxygen supplementation devices**			
Low-flow nasal cannula	310 (65.5%)	154 (66.4%%)	156 (64.7%)
High-flow nasal cannula	112 (23.7%)	52 (22.4%)	60 (24.9%)
**Treatments**			
Remdesivir	325 (68.7%)	157 (67.7%)	168 (69.7%)
Steroids	465 (98.3%)	231 (99.6%)	234 (97.1%)
LMWH	450 (95.1%)	222 (95.7%)	228 (94.6%)

LMWH: low-molecular-weight heparin

© 2021 Menichetti F et al. *JAMA Network Open.*

eTable 4. Characteristics of the Patients at the Baseline in the ITT Population

	Total No (%)	Convalescent plasma No (%)	Standard therapy No (%)
	**n=483**	**n=243**	**n=240**
**Age**, median [IQR]-yrs	64.0 [54.0-74.0]	63.0 [54.0-74.0]	65.0 (55.0-74.0]
**Gender**			
Female	173 (35.8%)	87 (35.8%)	86 (35.8%)
Male	310 (64.2%)	156 (64.2%)	154 (64.2%)
**Race/Ethnicity**			
White	446 (92.3%)	224 (92.2%)	222 (92.5%)
Non-white[a]	11 (2.3%)	5 (2.1%)	6 (2.5%)
Asian	8 (1.7%)	3 (1.2%)	5 (2.1%)
Others	17 (3.5%)	10 (4.1%)	7 (2.9%)
Missing	1 (0.2%)	1 (0.4%)	0 (0.0%)
**BMI**, median [IQR]	26.0 [24.1-28.6]	26.0 [23.9-28.1]	26.0 [24.2-29.2]
**Time from onset of symptoms**, median [IQR] - days	7.0 [5.0-9.0]	7.0 [4.0-9.0]	7.0 [5.0-9.0]
**Comorbidities**			
None	101 (20.9%)	51 (21.0%)	50 (20.8%)
Hypertension	184 (37.9%)	86 (35.8%)	97 (39.9%)
Type 2 diabetes	94 (19.5%)	49 (20.4%)	45 (18.5%)
COPD	28 (5.8%)	14 (5.8%)	14 (5.8%)
Chronic renal failure	23 (4.8%)	7 (2.9%)	16 (6.6%)
Solid cancer	17 (3.5%)	10 (4.2%)	7 (2.9%)
Congestive heart failure	11 (2.3%)	5 (2.1%)	6 (2.5%)
**Smoking**			
None	320 (66.3%)	159 (65.4%)	161 (67.1%)
Previous tobacco use	107 (22.2%)	59 (24.3%)	48 (20.0%)
Current tobacco use	15 (3.1%)	6 (2.5%)	9 (3.8%)
Missing	41 (8.5%)	19 (7.8%)	22 (9.2%)
**Disease severity**, median [IQR]			
PaO2/FiO2	273.0 [238.0-307.0]	266.0 [240.0-309.0]	277.0 [236.5-305.5]
SOFA score[1]	2.0 [2.0-3.0]	2.0 [2.0-3.0]	2.0 [2.0-2.5]
**Previous treatment**			
Remdesivir	13 (2.7%)	8 (3.3%)	5 (2.1%)
Corticosteroids	99 (20.5%)	52 (21.4%)	47 (19.6%)
LMWH	93 (19.3%)	46 (18.9%)	47 (19.6%)

[1]missing data for 110 patients (52 CP+ST; 58 ST)

[a] Race: non-white included: Black or African America.

CP: convalescent plasma; ST: standard therapy; No: number of patients; IQR: interquartile range; COPD: chronic obstructive pulmonary disease; BMI: body-mass index; SOFA: Sequential Organ Failure Assessment; LMWH: low-molecular-weight heparin

© 2021 Menichetti F et al. *JAMA Network Open.*

eTable 5. Primary End Point in the ITT Population

Analysis Population	Convalescent Plasma + Standard Therapy	Standard Therapy	OR (95% CI)[b]	p
	n/No (%)	n/No (%)		
ITT[a]	60/237 (25.3%)	67/240 (27.9%)	0.88 (0.58-1.31)	.52

© 2021 Menichetti F et al. *JAMA Network Open.*

eTable 6. Primary End Point in the mITT Population According to Lag From Onset of Symptoms to Convalescent Plasma (CP) Infusion

Lag onset of symptoms – CP infusion	Primary Endpoint n/No (%)	OR (95% CI)	p value
0-5 days	12/57 (21.0%)	0.67 (0.33-1.34)	.23
6-10 days	42/136 (30.9%)	1.15 (0.72-1.82)	.56
11+ days	5/37 (13.5)	0.40 (0.15-1.07)	.07

* Data available for 230/231 patients in th mITT population

© 2021 Menichetti F et al. *JAMA Network Open.*

eTable 7. Secondary End Points in the mITT Population

Secondary endpoints	Convalescent Plasma + Standard Therapy n/No (%)	Standard Therapy n/No (%)	p value
	**n=232**	**n=241**	
30-day mortality[a]	14/231 (6.1%)	19/240 (7.9%)	.43
Mechanical ventilation or death[b]	25/230 (10.9%)	25/240 (10.4%)	.87
Virological cure[c]	14/199 (7.0%)	13/209 (6.2%)	.93
	**Median [IQR]**		
Time from hospitalization to discharge, in days[d]	12 [7-23]	13 [7-25]	.73

[a]data not available for 2 patients (1 Convalescent Plasma + Standard Therapy and 1 Standard Therapy)
[b]data not available for 3 patients (2 Convalescent Plasma + Standard Therapy and 1 Standard Therapy)
[c] two consecutive nasopharyngeal swabs resulting negative; data not available for 65 patients (33 Convalescent Plasma + Standard Therapy and 32 Standard Therapy)
[d]data not available for 5 patients (3 Convalescent Plasma + Standard Therapy and 2 Standard Therapy)
n: number of patients reaching the endpoint; No: number of patients, IQR: interquartile range.
Comparison of categorical variables performed with log-rank; comparison of continous variables performed with the Mann-Whitney test.

© 2021 Menichetti F et al. *JAMA Network Open.*

eFigure 1. Primary End Point Outcome According to Trial Group and Clinical Sites in the mITT Population

Clinical sites*	CP n/N(%)	CP+ST n/N(%)	Odds Ratio with 95% CI	p**
01	14/58 (24.1%)	16/60 (26.7%)	0.88 [0.38, 2.01]	0.753
02	19/46 (41.3%)	9/47 (19.1%)	2.97 [1.17, 7.56]	0.022
03	5/27 (18.5%)	7/27 (25.9%)	0.65 [0.18, 2.38]	0.514
04	3/15 (20.0%)	2/15 (13.3%)	1.63 [0.23, 11.46]	0.626
05	2/11 (18.2%)	7/14 (50.0%)	0.22 [0.03, 1.42]	0.112
06	1/9 (11.1%)	4/9 (44.4%)	0.16 [0.01, 1.83]	0.139
07	2/8 (25.0%)	1/10 (10.0%)	3.00 [0.22, 40.93]	0.410
08	0/9 (0.0%)	0/9 (0.0%)	1.00 [0.02, 55.80]	/
09	1/6 (16.7%)	4/7 (57.1%)	0.15 [0.01, 2.05]	0.155
10	0/4 (0.0%)	3/8 (37.5%)	0.17 [0.01, 4.35]	0.491
11	3/6 (50.0%)	1/5 (20.0%)	4.00 [0.27, 60.32]	0.317
12	2/5 (40.0%)	2/5 (40.0%)	1.00 [0.08, 12.56]	1.000

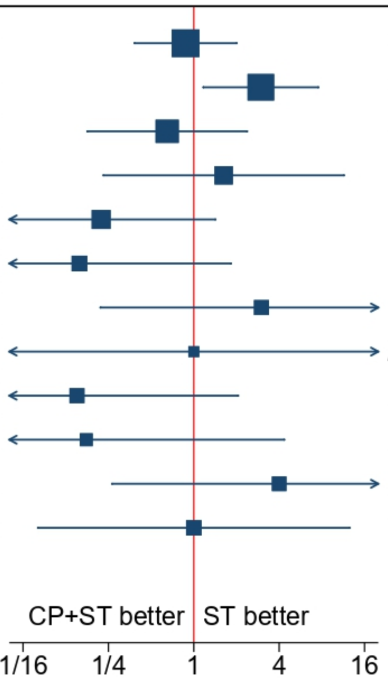

* Remaining clinical sites (13-27) enrolling less than 10 patients are not shown. ** p value for clinical site 10 was calculated by Fisher's exact test

CP: convalescent plasma; ST: standard therapy; n: number of events; N: number of patients; CI: confidence interval

© 2021 Menichetti F et al. *JAMA Network Open.*

eFigure 2. Survival Curves at 30 Days of Secondary Outcomes in the mITT Population

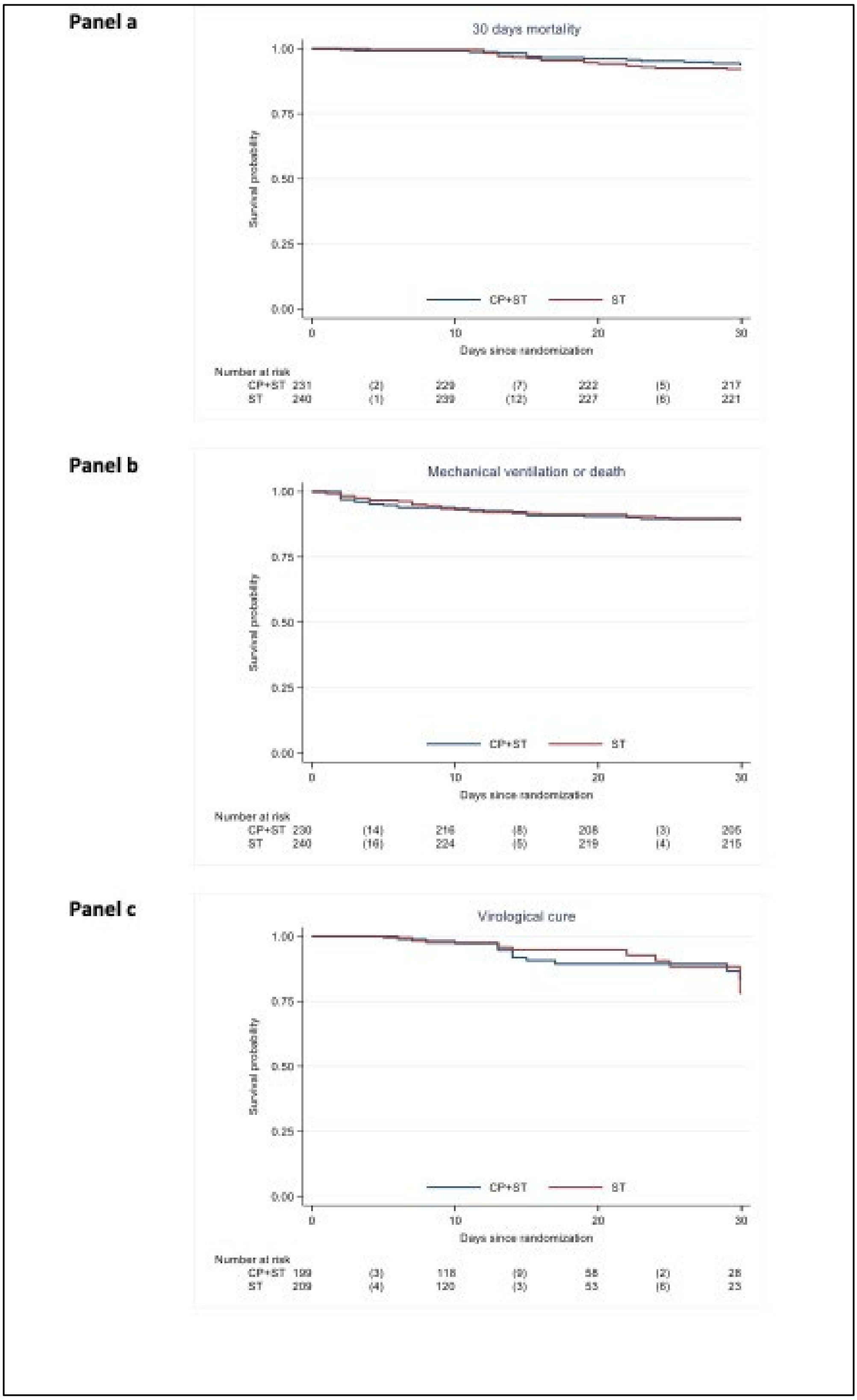

panel A: 30 days mortality, panel B: mechanical ventilation or death, panel C: virological cure, CP convalescent plasma; ST standard therapy.

© 2021 Menichetti F et al. *JAMA Network Open.*

“Number of events in the intervals 0-10 days, 10-20 days, and 20-30 days, respectively, are reported in brackets.”

© 2021 Menichetti F et al. *JAMA Network Open.*

eReferences

1. Wang R, Lagakos SW, Ware JH, Hunter DJ, Drazen JM. Statistics in medicine--reporting of subgroup analyses in clinical trials. N Engl J Med. 2007;357:2189-94
2. Bellan M, Patti G, Hayden E, et al. Fatality rate and predictors of mortality in an Italian cohort of hospitalized COVID-19 patients. Sci Rep. 2020;10:20731
3. European Medicines Agency (EMA). Reflection paper on methodological issues in confirmatory clinical trials planned with an adaptive design (CHMP/EWP/2459/02).

© 2021 Menichetti F et al. *JAMA Network Open.*
