# Supplementary material for: Effect of High-Titer Convalescent Plasma on Progression to Severe Respiratory Failure or Death in Hospitalized Patients With COVID-19 Pneumonia: A Randomized Clinical Trial
Source: JAMA Netw Open. 2021 Nov 29;4(11):e2136246. doi: 10.1001/jamanetworkopen.2021.36246 (PMC8630572; doi:10.1001/jamanetworkopen.2021.36246)
Supplement: Supplement 3. — Nonauthor Collaborators [file jamanetwopen-e2136246-s003.pdf]

Supplemental Online Content: Nonauthor Collaborators

*Indicates required information. Only first name, last name, and suffix will appear in PubMed.

***Group Name(s): TSUNAMI Study group**

| *First Name and Middle Initial(s) | *Last Name | *Suffix (eg, Jr, III) | Academic Degrees | Institution | Location (city, state/province, country) | Role or Contribution, eg, chair, principal investigator | Group (if more than 1 Group listed in the byline) and/or Subgroup (eg, Steering Committee) |
|---|---|---|---|---|---|---|---|
| Roberto | Palazzolo | | MD | Asst Valtellina e Alto Lario, Sondrio | Sondrio, Italy | investigator | TSUNAMI Study Group |
| Salvatore | Casari | | MD | Carlo Poma Hospital | Mantova, Italy | investigator | TSUNAMI Study Group |
| Alessandro | Occhionero | | MD | Carlo Poma Hospital | Mantova, Italy | investigator | TSUNAMI Study Group |
| Tiziana | Grazzini | | MD | Azienda Ospedaliera Universitaria Pisana | Pisa, Italy | assistant | TSUNAMI Study Group |
| Dina Leonarda | Silvestri | | MD | Azienda Ospedaliera Universitaria Pisana | Pisa, Italy | assistant | TSUNAMI Study Group |
| Mariacarla | Iorio | | MD | Azienda Ospedaliera Universitaria Pisana | Pisa, Italy | assistant | TSUNAMI Study Group |
| Andrea | Tosti | | MD | Department of Medicine, Clinic of Infectious Diseases, "Santa Maria della Misericordia" Hospital, University of Peru | Perugia, Italy | investigator | TSUNAMI Study Group |
| Daniela | Francisci | | MD | Department of Medicine, Clinic of Infectious Diseases, "Santa Maria della Misericordia" Hospital, University of Peru | Perugia, Italy | investigator | TSUNAMI Study Group |
| Cecilia | Becattini | | MD | Internal and Cardiovascular Medicine-Stroke Unit, University of Perugia | Perugia, Italy | investigator | TSUNAMI Study Group |
| Matteo | Pirro | | MD | Unit of Internal Medicine, Department of Medicine, University of Perugia, | Perugia, Italy | investigator | TSUNAMI Study Group |
| Mauro | Marchesi | | MD | Centro Regionale Sangue, Servizio Immunotrasfusionale, Azienda Ospedaliera di Perugia | Perugia, Italy | assistant | TSUNAMI Study Group |
| Sabrina | Bastianelli | | MD | Department of Medicine, Clinic of Infectious Diseases, "Santa Maria della Misericordia" Hospital, University of Peru | Perugia, Italy | investigator | TSUNAMI Study Group |
| Sara | Pierucci | | MD | Department of Medicine, Clinic of Infectious Diseases, "Santa Maria della Misericordia" Hospital, University of Peru | Perugia, Italy | investigator | TSUNAMI Study Group |
| Chiara | Busti | | MD | Department of Medicine, Clinic of Infectious Diseases, "Santa Maria della Misericordia" Hospital, University of Peru | Perugia, Italy | investigator | TSUNAMI Study Group |
| Antonella | Mencacci | | MD | Microbiology and Clinical Microbiology, Department of Medicine and Surgery, University of Perugia | Perugia, Italy | assistant | TSUNAMI Study Group |
| Silvia | Bozza | | MD | Microbiology and Clinical Microbiology, Department of Medicine and Surgery, University of Perugia | Perugia, Italy | assistant | TSUNAMI Study Group |
| Barbara | Camilloni | | MD | Microbiology and Clinical Microbiology, Department of Medicine and Surgery, University of Perugia | Perugia, Italy | assistant | TSUNAMI Study Group |
| Valentina | Annoni | | MD | Viareggio Hospital | Viareggio, Italy | investigator | TSUNAMI Study Group |
| Chiara | Bellotto | | MD | Viareggio Hospital | Viareggio, Italy | investigator | TSUNAMI Study Group |
| Adriano | Cioppi | | MD | Viareggio Hospital | Viareggio, Italy | investigator | TSUNAMI Study Group |
| Giorgia | Querci | | MD | Viareggio Hospital | Viareggio, Italy | investigator | TSUNAMI Study Group |
| Giacomo | Ciusa | | MD | Infectious Diseases Unit, Azienda Ospedaliero-Universitaria di Modena | Modena, Italy | investigator | TSUNAMI Study Group |
| Michela | Tassara | | MD | Immunohematology and Transfusion Medicine, IRCCS Ospedale San Raffaele, Milan | Milan, Italy | assistant | TSUNAMI Study Group |
| Anna | Danise | | MD | Infectious Diseases, IRCCS Ospedale SanRaffaele, Università Vita-Salute San Raffaele, Milan | Milan, Italy | investigator | TSUNAMI Study Group |
| Silvia | Chigiotti | | MD | Division of Infectious Diseases, Hospital of Grosseto | Grosseto, Italy | investigator | TSUNAMI Study Group |
| Giovanna | Morelli | | MD | Infectious Disease Unit, Hospital of Lucca | Lucca, Italy | investigator | TSUNAMI Study Group |
| Micaela | Meini | | MD | Infectious Disease Unit, Hospital of Lucca | Lucca, Italy | investigator | TSUNAMI Study Group |
| Valentina | Galfo | | MD | Infectious Disease Unit, Department of Clinical and Experimental Medicine, Azienda Ospedaliera Universitaria Pisana, University of Pisa | Pisa, Italy | investigator | TSUNAMI Study Group |
| Simone | Ferranti | | MD | Infectious Disease Unit, Azienda Ospedaliera Universitaria Pisana | Pisa, Italy | investigator | TSUNAMI Study Group |
| Enrico | Tagliaferri | | MD | Infectious Disease Unit, Azienda Ospedaliera Universitaria Pisana | Pisa, Italy | investigator | TSUNAMI Study Group |
| Riccardo | Iapoce | | MD | Infectious Disease Unit, Azienda Ospedaliera Universitaria Pisana | Pisa, Italy | investigator | TSUNAMI Study Group |
| Chiara | Barbieri | | PhD | Infectious Disease Unit, Department of Clinical and Experimental Medicine, Azienda Ospedaliera Universitaria Pisana, University of Pisa | Pisa, Italy | investigator | TSUNAMI Study Group |

Supplemental Online Content: Nonauthor Collaborators

*Indicates required information. Only first name, last name, and suffix will appear in PubMed.

| ***First Name and Middle Initial(s)** | ***Last Name** | ***Suffix (eg, Jr, III)** | Academic Degrees | Institution | Location (city, state/province, country) | Role or Contribution, eg, chair, principal investigator | Group (if more than 1 Group listed in the byline) and/or Subgroup (eg, Steering Committee) |
|---|---|---|---|---|---|---|---|
| Arianna | Forniti | | MD | Infectious Disease Unit, Department of Clinical and Experimental Medicine, Azienda Ospedaliera Universitaria Pisana, University of Pisa | Pisa, Italy | investigator | TSUNAMI Study Group |
| Claudio | Caroselli | | MD | Infectious Disease Unit, Department of Clinical and Experimental Medicine, Azienda Ospedaliera Universitaria Pisana, University of Pisa | Pisa, Italy | investigator | TSUNAMI Study Group |
| Stefano | Verdenelli | | MD | Infectious Disease Unit, Azienda Ospedaliera Universitaria Pisana | Pisa, Italy | investigator | TSUNAMI Study Group |
| Fabio | Monzani | | MD | Geriatric Unit, Azienda Ospedaliera Universitaria Pisana | Pisa, Italy | investigator | TSUNAMI Study Group |
| Paola | Mazzetti | | MD | Division of Virology, Pisa University Hospital | Pisa, Italy | assistant | TSUNAMI Study Group |
| Giovanna | Moscato | | MD | Division of Laboratory Analysis, Pisa University Hospital | Pisa, Italy | assistant | TSUNAMI Study Group |
| Francesco | Barchiesi | | MD | Infectious Diseases Unit, Pesaro Hospital | Pesaro, Italy | investigator | TSUNAMI Study Group |
| Mauro | Andreotti | | MSC | National Center for Global Health, Istituto Superiore di Sanità | Rome, Italy | Scientific investigator | TSUNAMI Study Group |
| Fausto | Baldanti | | MD | Laboratory of Virology and Microbiology, San Matteo Hospita | Pavia, Italy | Senior scientific investigator | TSUNAMI Study Group |
| Andrea | Binelli | | MSC | National Center for Global Health, Istituto Superiore di Sanità | Rome, Italy | Scientific investigator | TSUNAMI Study Group |
| Maria R | Capobianchi | | MD, PhD | Laboratory of Virology, Istituto Nazionale per le Malattie Infettive (INMI) “L. Spallanzani” | Rome, Italy | Senior scientific investigator | TSUNAMI Study Group |
| Roberto | Da Cas | | CSC | National Center for Drug Research and Evaluation, Istituto Superiore di Sanità | Rome, Italy | Scientific investigator | TSUNAMI Study Group |
| Daniela | Di Sevo | | MSC | Gimema Foundation | Rome, Italy | Clinical monitor | TSUNAMI Study Group |
| Paola | Fazi | | MD PhD | Gimema Foundation | Rome, Italy | GIMEMA Data Center Director | TSUNAMI Study Group |
| Cinzia | Gasparrini | | Secretary | Department of Neuroscience, Istituto Superiore di Sanità | Rome, Italy | Assistant | TSUNAMI Study Group |
| Ilaria | Ippoliti | | MSC | National Center for Drug Research and Evaluation, Istituto Superiore di Sanità | Rome, Italy | Scientific | TSUNAMI Study Group |
| Alessandra | Mancino | | MSC | Gimema Foundation | Rome, Italy | Scientific project manager | TSUNAMI Study Group |
| Francesca | Menniti Ippolito | | PhD | National Center for Drug Research and Evaluation, Istituto Superiore di Sanità | Rome, Italy | Senior Scientific investigator | TSUNAMI Study Group |
| Francesca | Paoloni | | Statisticia n | Gimema Foundation | Rome, Italy | Data Base project manager | TSUNAMI Study Group |
| Paola | Ruggeri | | Secretary | National Center for Drug Research and Evaluation, Istituto Superiore di Sanità | Rome, Italy | Assistant | TSUNAMI Study Group |
| Arianna | Rughini | | MSC | Gimema Foundation | Rome, Italy | Clinical monitor | TSUNAMI Study Group |
| Emanuela | Salvi | | Secretary | National Center for Drug Research and Evaluation, Istituto Superiore di Sanità | Rome, Italy | Assistant | TSUNAMI Study Group |
| Valeria | Sargentini | | PhD | Gimema Foundation | Rome, Italy | Data manager | TSUNAMI Study Group |
| Maria P | Trotta | | MD | Agenzia Italiana del Farmaco | Rome, Italy | Advisor | TSUNAMI Study Group |
| Marco | Vignetti | | MD | Gimema Foundation | Rome, Italy | Advisor | TSUNAMI Study Group |
