# Supplementary material for: Effect of High-Titer Convalescent Plasma on Progression to Severe Respiratory Failure or Death in Hospitalized Patients With COVID-19 Pneumonia: A Randomized Clinical Trial
Source: JAMA Netw Open. 2021 Nov 29;4(11):e2136246. doi: 10.1001/jamanetworkopen.2021.36246 (PMC8630572; doi:10.1001/jamanetworkopen.2021.36246)
Supplement: Supplement 4. — Data Sharing Statement [file jamanetwopen-e2136246-s004.pdf]

Data Sharing Statement

Menichetti. Effect of High-Titer Convalescent Plasma on Progression to Severe Respiratory Failure or Death in Hospitalized Patients With COVID-19 Pneumonia. *JAMA Netw Open*. Published November 29, 2021. doi:10.1001/jamanetworkopen.2021.36246

Data
Data available: Yes
Data types: Deidentified participant data
How to access data: Database available from GIMEMA
When available: With publication

Supporting Documents
Document types: Statistical/analytic code
How to access documents: Documents available from GIMEMA
When available: With publication

Additional Information
Who can access the data: esearchers whose proposed use of the data has been approved
Types of analyses: for a specified purpose
Mechanisms of data availability: after approval of a proposal and with a signed data access agreement
